# Assembly of chromosome-scale contigs by efficiently resolving repetitive sequences with long reads

**DOI:** 10.1038/s41467-019-13355-3

**Published:** 2019-11-25

**Authors:** Huilong Du, Chengzhi Liang

**Affiliations:** 1 State Key Laboratory of Plant Genomics, Institute of Genetics and Developmental Biology, Innovation Academy for Seed Design, Chinese Academy of Sciences, 1 Beichen West Road No. 2, Beijing, 100101 China; 2 University of Chinese Academy of Sciences, Beijing, 100049 China

**Keywords:** Genome assembly algorithms, Software, Genome, DNA sequencing

## Abstract

The abundant repetitive sequences in complex eukaryotic genomes cause fragmented assemblies, which lose value as reference genomes, often due to incomplete gene sequences and unanchored or mispositioned contigs on chromosomes. Here we report a genome assembly method HERA, which resolves repeats efficiently by constructing a connection graph from an overlap graph. We test HERA on the genomes of rice, maize, human, and Tartary buckwheat with single-molecule sequencing and mapping data. HERA correctly assembles most of the previously unassembled regions, resulting in dramatically improved, highly contiguous genome assemblies with newly assembled gene sequences. For example, the maize contig N50 size reaches 61.2 Mb and the Tartary buckwheat genome comprises only 20 contigs. HERA can also be used to fill gaps and fix errors in reference genomes. The application of HERA will greatly improve the quality of new or existing assemblies of complex genomes.

## Abstract (web summary)

Repetitive sequences in complex eukaryote genomes can cause fragmented assemblies with incomplete gene sequences and unanchored or mispositioned contigs. Here, the authors report HERA, a method to improve genome assemblies by efficiently resolving repeats using single-molecule sequencing data.

## Introduction

Assembly of highly continuous and complete genome sequences is crucial for identifying structural variations, as well as for gene mapping and cloning. Complex eukaryotic genomes contain a large number of repetitive sequences which complicates the genome assembly process^1^. In the past decade, next generation sequencing assisted the assembly of hundreds of draft animal and plant genomes. Improvements in sequencing read lengths to tens of kb by single-molecule sequencing (SMS) technologies from Pacific Biosciences^2^ (PacBio) and most recently from Oxford Nanopore^3^ have enabled the assembly of many complex eukaryotic genomes. However, these assemblies are still fragmented and generate incomplete draft genomes usually consisting of thousands of contigs with many unresolved regions caused by segmentally duplicated repeats or other complex repeats^4–6^. The missing repeats in a genome assembly may have important functional implications. For example, segmental duplications account for ~5% of the human genome and are associated with structural variations and genetic diseases, as well as showing large impact on human evolution and adaptation^7–9^.

The SMS long reads are currently assembled using String Graph (SG)^10^ or -like assemblers such as FALCON^11^ and Celera Assembler^12^ (CA). CA represents the traditional Overlap-Layout-Consensus (OLC) assemblers and uses overlap graph^13^ to store reads and sequence overlaps between them. OLC is now used in several genome assemblers including PBcR^14^, CANU^15^, and MECAT^16^. A string graph^10^ reduces an overlap graph using a transitive reduction step to collapse identical or similar sequences. Tiling paths of reads are identified and joined together to form contigs, but multiple copies of similar repeats are also compressed. The approach assembles unique sequences reliably but repeats longer than the read length lead to branching paths and thus form fragmented contigs. As a result, the current assemblers can generate a set of well-assembled unique sequences, but many of these sequences are fragmented in the form of very short contigs due to their surrounding by unassembled regions. Therefore, accurate assembly and resolution of large repeats and highly similar haplotypes in heterozygous genomes still remains a major challenge in genome assembly efforts.

Genome assembly projects often generate chromosome-scale pseudomolecules to anchor the assembled contigs/scaffolds with genetic maps or Hi-C data^17^. However, the approaches often leave many misordered and misoriented contigs in pseudomolecules, as well as unanchored orphan contigs. The genome maps from BioNano Genomics^18^ covering megabases (Mb) in length can be used to link contigs to hybrid scaffold sequences. However, the method cannot improve the contig lengths and often leaves unfilled gaps of up to hundreds of kb and many unmapped contigs due to lack of labeling enzyme recognition sites.

To improve the quality of the assembled genomes, one key step is to assemble the unassembled regions into contiguous sequences by focusing on repeat resolution. In our previous work^5^, we applied a combination of experimental and computational method by using fosmid clone sequencing to assist the assembly of SMS sequencing data. However, the relatively high cost of construction and sequencing of a fosmid library inhibits the wide application of this method to a large number of genomes. On the other hand, several algorithms have been developed in the past to assemble the highly repetitive regions^19,20^. However, these methods have not been optimized to explore the full potentials of SMS long reads in assembling repeats in complex genomes. Recently, a method SDA was developed based on polyploid phasing^21^ to accurately assemble the different paralogs of many segmental duplications in human genome^22^. However, the usage of SDA depends on reference sequences and the majority of the assembled paralogs were not linked to their flanking unique sequences, i.e., still remaining orphan contigs.

Here we report a highly efficient genome assembly method using SMS data to resolve repeats called HERA (Highly Efficient Repeat Assembly), which enables the assembly of highly contiguous genomes by assembling each individual repeat separately and correctly connecting it to its true flanking sequences. With the help of BioNano genome maps and chromosomal anchoring information, HERA can generate ultra-long, even chromosome-scale, contigs. We test the method on the genomes of rice, maize, human, and Tartary buckwheat, and show that it dramatically improves the sequence contiguity of the assemblies produced by existing assemblers. We generate a high-quality reference genome for maize and Tartary buckwheat with filled gaps and error corrections. The application of HERA will greatly improve the quality of new or existing assemblies of complex genomes.

## Results

### Overview of HERA algorithm

The goal of HERA is to assemble the missing DNA sequences between a set of existing DNA sequences in a genome. The locations of these existing DNA sequences on chromosome can be either known or undetermined but they are assumed to be distributed relatively evenly. In this work, HERA utilizes the contigs generated from other assemblers to delimit the genome regions to be assembled. We call two contigs adjacent if there are no other contigs being present between them on chromosome. HERA works by assembling as many missing regions as possible to connect the adjacent contigs into longer contigs. This process includes two interconnected questions: (1) which pairs of contigs are adjacent and (2) which pairs of contigs can the regions between be correctly assembled? The answer to either of them will help answer the other.

HERA constructs an undirected overlap graph consisting of two types of nodes: anchoring nodes to represent pre-assembled contigs and read nodes to represent SMS reads. For simplicity, we use the term sequence to represent a DNA sequence of any length throughout this work. Two sequences are called overlapping if they contain similar sequences (Supplementary Fig. 1a). Since each sequence has two ends, every node in the overlap graph is assigned with two ends which emanate two separate groups of incident edges, i.e., the nodes have two directions of in and out which are exchangeable in graph traversal. We do not explicitly specify the node end (sequence direction) in the following text unless it is necessary. At present, the noisy raw SMS reads are self-corrected by using the self-correction module in CANU. The overlaps between all sequences including the contigs and the corrected reads are identified by using a sequence aligner such as BWA^23^. Using overlap graph, the key issue in HERA is to maximize the number of identified anchoring node pairs that represent adjacent contigs.

**Fig. 1:**
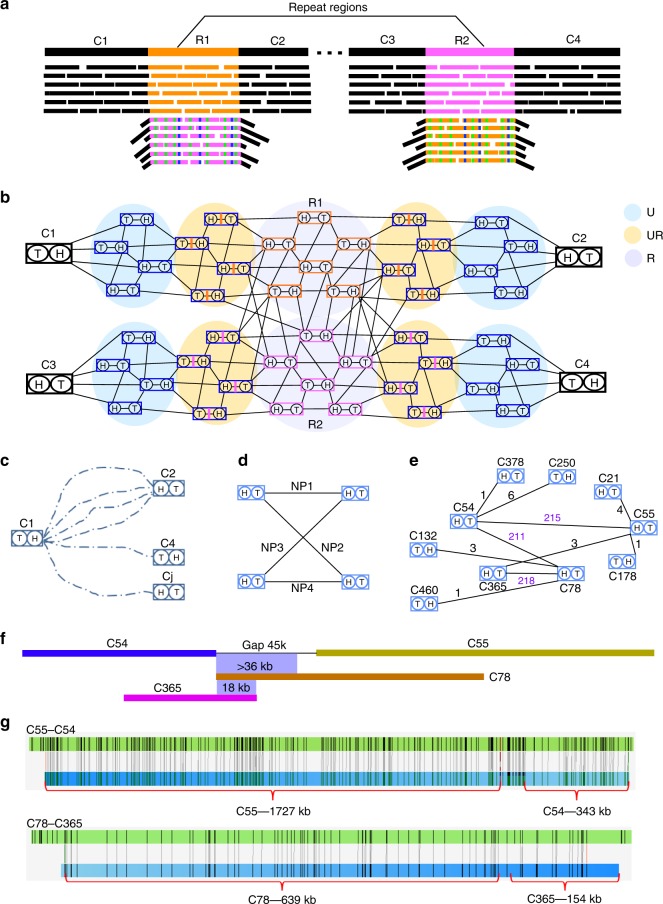
Overview of HERA. **a** Two copies of repeats (R1 and R2) are similar to each other but they also contain sequence variations which can be found in the reads originating from them. The alignments of junction reads (across the boundary between repeat and unique sequences) to a different repeat copy form overhangs of unaligned, unique sequences. **b** A subgraph of an overlap graph corresponding to the genome segments and sequencing reads shown in (**a**). The sequencing reads can be classified into three types: unique reads (U), repeat reads (R) and junction reads (UR). **c** The path extension from contig end C_1_^h^ can reach a set of other contig ends, which include C_2_^h^, the true target, and C_4_^t^, the false target, and possibly others (C_j_^h^) from background noise. **d** A connection graph showing the number of paths (NP) between each pair of contig nodes. **e** A subgraph of a connection graph with examples of conflicting connections. The conflicting indices of two contig ends were: CI_54_^t^ = 211/215 = 0.98; CI_78_^h^ = 211/218 = 0.97. These conflicting connections can be resolved because the number of paths between C_365_^t^-C_55_^h^ was very small, so that C_78_^h^-C_365_^t^ can be connected first. **f** Sequence alignments showing a fragment of at least 36 kb in C_78_ being similar to the connecting sequence between C_54_^t^ and C_55_^h^ and 18 kb of highly similar sequence in C_365_^t^ overlapping C_78_^h^. **g** The alignments to BioNano genome maps confirmed that the connections of C_54_^t^-C_55_^h^ and C_78_^h^-C_365_^t^ were correct.

We used an example in Fig. 1 to illustrate the HERA algorithm, which is fully described in Methods. Assume that two similar segmentally duplicated repeat copies R1 and R2 are located between two pairs of unique sequences C_1_/C_2_ and C_3_/C_4_, respectively (Fig. 1a). Note that the relationship between the four sequences is not known beforehand. The part of overlap graph is shown in Fig. 1b. For simplicity we use symbols C1–C4 to also represent the nodes. Clearly there must be a path from C1 to C2 in the overlap graph under high sequencing depth. Further, an anchoring node such as C1 (in one direction) is often connected to multiple anchoring nodes (such as C2 and C4) due to the presence of repeats (Fig. 1b).

By graph traversal, HERA builds a set of high-scoring paths (representing tiling read paths) from every anchoring node to a set of ending anchoring nodes (Fig. 1c). Further, HERA constructs a new graph, which is here called a connection graph, consisting of only the anchoring nodes from the overlap graph. The edge length of the connection graph is set to the number of high-scoring paths between each pair of anchoring nodes (Fig. 1d). With the help of connection graph, HERA identifies the adjacent sequences such as C_1_/C_2_ and C3/C_4_ and finishes each local assembly by using a consensus sequence to connect each adjacent contig pair. Obviously, if one anchoring node is connected to only one other anchoring node, then the genome region is considered to be a unique sequence whose assembly is trivial.

To assemble segmentally duplicated sequences, HERA fully takes advantage of the sequence variations between different repeat copies to distinguish them from each other (Fig. 1a and Supplementary Fig. 1b, c). Note that the read overlapping a contig end is usually either a repetitive sequence or a junction sequence consisting of partial unique sequence and partial repetitive sequence. During the path extension process toward the repetitive direction, the overlapping reads originating from the same repeat copy usually have a higher chance of being selected than those from different repeat copies due to the higher sequence similarity of the former. This leads to the separation of most repeat copies with sequence identity below a pre-defined threshold (currently it is ~99%), which is dependent on the nucleotide accuracy in the reads.

For two nearly identical repeat copies (e.g., sequence identity >99.5%) (Supplementary Fig. 1d), it is expected that many reads originating from them will be selected rather randomly during the path extension process. This leads to two incident edges of a node end with similar length in the connection graph, i.e., conflicting connections (Fig. 1e, f). HERA does not connect a contig end to other contigs if it has conflicting connections. As a result, HERA ensures that repetitive sequences be assembled with its true flanking sequences to minimize the number of chimeric contigs and the error rate in the assembled sequences. There are several ways to resolve the conflicts: first, by removing one of the connected anchoring nodes when it can be more confidently connected to another anchoring node (Fig. 1e); Second, by using BioNano genome maps or mate-pair scaffolding information to identify the correct pair (Fig. 1g); Third, by using the chromosomal grouping information derived from genetic map or Hi-C data to identify the correct pair.

**Fig. 2:**
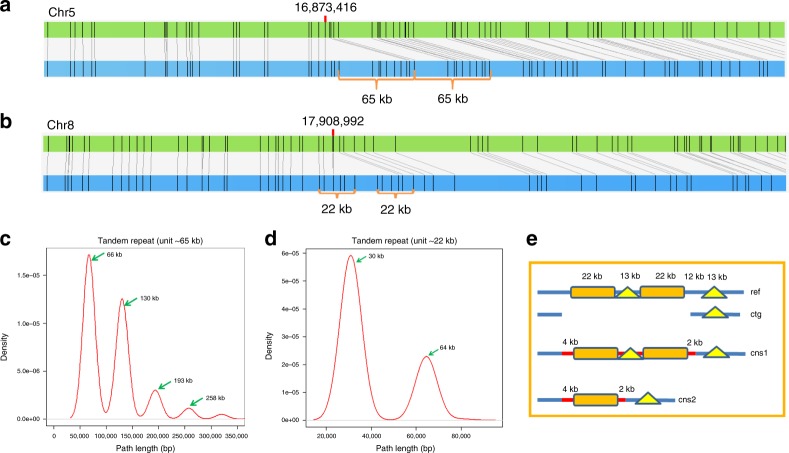
An illustration of identifying tandemly repetitive sequences by HERA. **a** A tandemly repetitive sequence on chromosome 5 of R498 with a unit length of 65 kb. The upper green horizontal bar represents the assembled sequence lacking a unit and the lower blue bar represents the BioNano map. **b** A repetitive sequence on chromosome 8 of R498 with a unit length of 22 kb. **c** The length distribution of HERA generated tiling paths for the repeat shown in (**a**). The paths are divided into several clusters and the distances between adjacent peaks are 65 kb which matched the repeat unit length in (**a**). The second peak represents the whole region of two repeat units (130 kb). **d** The length distribution of HERA generated tiling paths for the repeat in (**b**). The paths are divided into two clusters and the distance between the two peaks is around 35 kb. **e** The schematic representation of the repeat region in (**b**). In this region, there are two highly similar repeat units of 22 kb (rectangle) being separated by one of the two dissimilar repeat units of 13 kb (triangle). Ref, the full repeat region; ctg, the flanking sequences to be connected; cns1 and cns2, excluding the flanking sequences shown in ctg, correspond to the second and the first peak in (**d**), respectively.

In addition to assemble unique sequences and segmentally duplicated repeats, HERA is also able to identify tandem repeats or complex repeats (Fig. 2a, b) by identifying multiple peaks in the path length distribution plot (Fig. 2c, d) and select the correct one as the assembled sequence (Fig. 2e). HERA is also extremely useful to fill known gaps and fix errors in the scaffolds or pseudomolecules assembled with genome maps, genetic maps or Hi-C data. On one hand, the known gap length is helpful for selecting the correct path. On the other hand, the connection information (number of paths and the average overlap sequence similarity in the paths) between contigs is helpful to determine the order and orientation of the contigs in pseudomolecules.

To fully explore the potential of HERA in assembling highly contiguous sequences, we incorporated HERA into an assembling pipeline which integrates single-molecule, real time (SMRT) sequencing data, BioNano genome maps and contig grouping information based on Hi-C clustering, genetic maps, or reference genomes (Supplementary Fig. 2). We assembled the genomes of rice, maize, human, and Tartary buckwheat (Table 1, Supplementary Tables 1, 2) with the pipeline to evaluate the performance of HERA.

**Table 1:** The summary of genome assemblies.

Genome	Method	Seq Num	N50 (Mb)	Max Len (Mb)	Total Len (Mb)
R498	CANU	811	1.31	5.43	402.5
	CANU + HERA	206	13.24	25.88	399.2
	BioNano map	453	1.22	5.78	406.1
	BioNano + CANU^a^	105	5.67	18.25	388.9
	BioNano + HERA^a^	32	17.51	32.2	390.2
	BioNano + HERA + GF	89	14.42	30.03	391.1
	On Chromosome	73	14.42	30.03	390.5
	R498_HERA1^b^	40	15.38	30.03	391.6
B73	RefGen_v4 (PBcR)	2,790	1.28	7.26	2106.3
	PBcR + HERA	416	31.53	121.28	2118.2
	BioNano map	1271	2.51	12.45	2079.7
	BioNano + PBcR^a^	319	10.2	45.88	2060.3
	BioNano + HERA^a^	68	107.5	194.6	2110
	BioNano + HERA + GF	130	61.2	142.5	2105.8
	B73_HERA1^c^	86	61.2	142.5	2103.9
HX1	HX1_FALCON	2,710	8.33	38.18	2873.2
	FALCON + HERA	850	32.53	109.81	2840.3
	BioNano map	2,487	1.68	11.41	2890.7
	BioNano + FALCON^a^	325	24.05	83.66	2724.4
	BioNano + HERA + GF	1,518	54.41	109.81	2871.3
	HX1_HERA1^c^	815	54.41	109.81	2841.7
Pinku1	CANU	839	1.1	10.83	452.1
	PBcR	6,033	0.45	2.11	587.7
	PBcR + HERA	48	22.24	43.19	453.4
	BioNano map	374	1.71	6.85	461.3
	BioNano + PBcR^a^	550	5.43	15.04	451.9
	BioNano + HERA^a^	22	51.77	61.99	453.7
	BioNano + HERA + GF	30	27.85	49.83	453.2
	Pinku1_HERA1^c^	20	51.77	62.08	453.5

*Seq Num* the total number of contigs or scaffolds, BioNano maps do not have sequences, *+GF* with gap filling, *On Chromosome* the HERA contigs anchored on nuclear genome chromosomes

^a^Hybrid scaffolds included unfilled gaps

^b^With gap filling after anchoring on chromosomes

^c^Only the contigs anchored on chromosomes were included here (no gap filling after anchoring on chromosomes). The unanchored sequences may include contaminations from other species. The sequences on chromosomes were corrected using Illumina short reads, which changed the sequence length

### Validation of HERA using rice R498 genome

First, we tested HERA using indica rice R498^5^ (Table 1). We used CANU to assemble the genome into 402.5 Mb sequences with a contig N50 size of 1.3 Mb. After HERA assembly, the contig N50 size was increased to 13.2 Mb. With the help of BioNano genome maps, which resolved a total of 61 conflicting connections, the contig N50 size was further increased to 14.4 Mb, with chromosome 8 assembled to a single contig. The non-rice genome sequences were filtered out by comparing them to the R498 reference genome^5^ (R498_ref1) and the remaining contigs formed pseudomolecules. After further gap filling, only 40 contigs were left with a contig N50 size of 15.38 Mb (R498_HERA1). The R498_HERA1 assembly contained approximately 1.5 Mb more sequences than R498_ref1. Comparison between the two assemblies showed that 95% of the newly increased sequences were centromeric or subtelomeric repeats. For example, R498_HERA1 contained a previously unidentified missing sequence of 387 kb in R498_ref1 located close to the centromere of chromosome 8 (Supplementary Fig. 3a). Notably, the CANU assembly contained several chimeric contigs which were easy to identify after HERA assembly due to the increased contig length, by comparison to genome maps or by using the genetic map we constructed previously^5^.

In R498_ref1, there were 14 known potential InDels (inserted or deleted sequences) >10 kb^5^. Using HERA to find paths in the overlap graph, we found that at least 8 of the InDels could be fixed using a minimum sequence identity of 97% as a cutoff during path extension (Supplementary Fig. 3b-g). Almost all of the regions were found to contain tandemly repetitive sequences with multiple peaks in their path length distribution plot. These results indicated that our assembly method for tandem repeats is valid.

HERA assembled 525 repeat regions with a total size of 6.4 Mb and a max length of 268.7 kb. Among the 495 (94.28%) regions that are covered by BioNano genome maps, we found only 19 (3.83%) containing InDels >10 kb, which set a max InDel error rate of 3.83% for the HERA assembly. As a comparison, we also found 20 InDels >10 kb in the CANU assembled contigs. Although we did not try to fix all of these potential errors, we believe that the majority of them can be fixed under the guidance of BioNano genome maps with known length based on our tests in the tandem repeat regions.

The nucleotide quality of all the HERA assembled sequences was validated with both corrected SMRT reads and Illumina short reads (Supplementary Fig. 4a–f) as well as rice BAC (bacterial artificial chromosome) sequences downloaded from GenBank nucleotide database. The short reads had a mapping ratio of 97.21% to the whole genome. The short reads covered 99.11% of the HERA assembled regions; as a comparison, they covered 99.45% of the rest of the genome. The mapping identity of the short reads on the HERA assembled regions is 99.67% (vs. 99.71% on the rest of the genome). The mapping identity and coverage of corrected SMRT reads in the CANU assembled regions are 98.84% and 99.94%, respectively, while they are 98.71% and 99.83% in the HERA assembled regions, respectively. These results suggest that the sequence quality of HERA assembled regions is almost the same as that of CANU assembled regions. Among the BACs downloaded from GenBank, we found eight BACs aligned to HERA assembled regions with a total length of 1,291,323 bp (Supplementary Table 3). These HERA and CANU assembled sequences were 481,929 bp and 809,394 bp, with an average sequence identity of 99.48% and 99.45% to the BAC sequences, respectively. These results further confirmed the high quality of the HERA assembled sequences.

### Improving maize B73 genome assembly

**Fig. 3:**
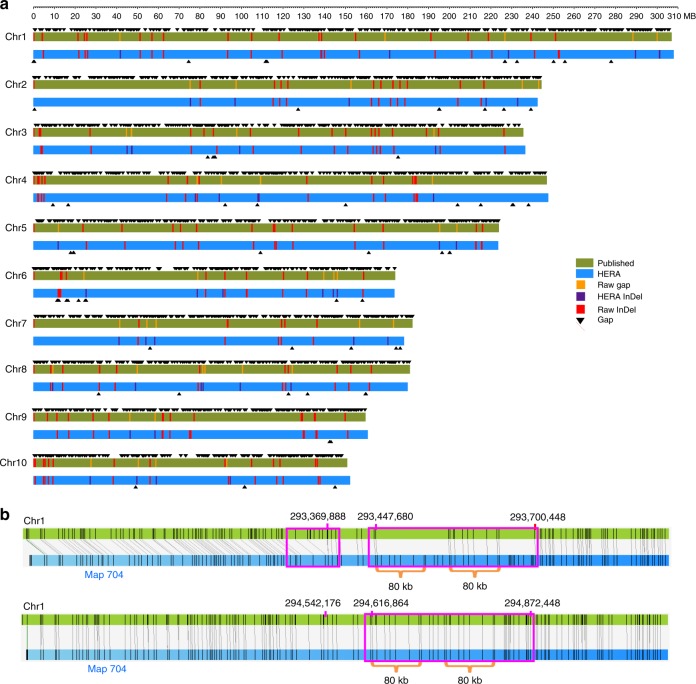
Comparison of maize B73 HERA assembly and RefGen_v4. **a** The comparison of HERA assembled B73 genome with the published B73 RefGen_v4. The top green horizontal bar represents RefGen_v4 and the bottom blue horizontal bar represents the HERA assembly. Each black triangle represents a sequence gap. The red vertical bars represent the >10 kb InDels that were present in the contigs of RefGen_v4. The purple vertical bars represent the >10 kb InDels introduced by HERA with the orange vertical bars showing the positions of the corresponding gaps in RefGen_v4. **b** An example of gap filling and sequence correction by HERA. The green horizontal bars represent the maize genome sequences and the blue horizontal bars represent BioNano maps. The upper panel is the alignment between RefGen_v4 and BioNano maps, and the lower panel is the alignment between the HERA assembly and BioNano maps. The gaps (right red box in the upper panel) in RefGen_v4 were filled with ‘N’s, which were correctly assembled by HERA. The inserted sequence in the left red box was not present in the HERA assembly.

To demonstrate the power of HERA in assembling more complex genomes, we improved the previously published maize B73 reference genome RefGen_v4^6^ (Table 1). The RefGen_v4 assembly contained many unfilled gaps (a total of 30.7 Mb of ‘N’s but the exact gap length is unknown) in the pseudomolecules (Supplementary Table 4), as well as a large number of small contigs (90.55 Mb in total length) that were not anchored on chromosomes. The HERA-improved assembly B73_HERA1 had a contig N50 size 61.2 Mb, a nearly 47x increase from 1.28 Mb. The longest contig in B73_HERA1 was 142.5 Mb, a 20-fold increase from 7 Mb. Owing to the improved contig length, the total length of the contig sequences anchored on chromosomes was increased from 2075.6 Mb to 2103.9 Mb (Supplementary Table 4), with only 2.8 Mb sequences remaining unanchored. The HERA assembled pseudomolecules contained only 76 gaps, a dramatic reduction from the 2,523 gaps in RefGen_v4 (Fig. 3a, b and Supplementary Table 4).

HERA assembled 2632 sequence regions, with a total length of 33.2 Mb and a maximum length of 276.7 kb, out of which 2,588 (98.33%) were covered and validated by BioNano genome maps (Supplementary Fig. 5a). We found that 47 (1.81%) HERA assembled sequences contained InDels >10 kb (Supplementary Table 5), most likely due to complex repeats (Supplementary Fig. 5b). As a comparison, the contigs in RefGen_v4 contained 140 InDels >10 kb (Supplementary Table 5). The gaps filled by HERA, with a length being up to ~586 kb (Supplementary Fig. 5c, d), included both missing sequences and unanchored sequences in RefGen_v4. The newly anchored sequences in the HERA assembly contained 815 of 837 genes not placed on chromosomes in RefGen_v4^6^. Meanwhile, the HERA assembled sequences also contained several important genes such as PWZ53301.1^24^, ACG34567.1^25^ and PWZ29282.1^24^ that were absent in RefGen_v4 (Supplementary Fig. 6a-c).

By assembling the short contigs into ultra-long contigs, HERA automatically corrected several inverted and misplaced sequences in RefGen_v4 (Supplementary Figs 5c and 7a, b). These errors were not identified using BioNano genome maps most likely due to the short contig length in RefGen_v4. These results also indicated that even after intensive curation, the reference genome can still contain structural errors other than InDels^26^. We did not fix the InDels originally present in the contigs of RefGen_v4 in the HERA assembled sequences we provided here (Supplementary Fig. 7c). To test HERA’s potential to fix these InDels, we randomly selected 37 of them and split the regions to form gaps with a length up to 126 kb. Among them, 31 (83.78%) could be correctly assembled as validated with genome maps (Supplementary Fig. 7d). These results indicated that HERA can be used as a curation tool to fix existing problems in reference genomes.

### Improving human HX1 genome assembly

Similarly, we applied HERA to improve the previously published human genome HX1_FALCON^4^ (Table 1). The HX1_HERA1 assembly had a contig N50 size 54.4 Mb, a tremendous increase from 8.3 Mb. The longest contig in HX1_HERA1 was 109.8 Mb, almost 3 times of the max length of 38 Mb in HX1_FALCON. Comparisons between HX1_HERA1, HX1_FALCON and human GRCh38 showed that several gaps in GRCh38 which remained open in HX1_FALCON could be filled in HX1_HERA1 (Supplementary Fig. 8a–f), and that GRCh38 contained some potential errors that could be fixed with the help of HERA (Supplementary Fig. 9a–c and Supplementary Table 6).

We examined the sequence quality of HERA assembled regions in HX1_HERA1 by comparing to 10 other genomes (including GRCh38) downloaded from GenBank nucleotide database, which has a contig N50 size of 16–57 Mb (Supplementary Table 7). We aligned the HERA assembled sequences along with a 50-kb flanking sequence on both sides in HX1_HERA1 to the 10 genomes. For each HERA assembled sequence in HX1_HERA1, we selected only the best matched location which included at least one flanking sequence aligned together in each genome. We found that 70–85% of the HERA sequences were covered by each of the 10 genomes with mapping identity of 98.97–99.30%, while the mapping identity of the flanking sequences was 98.40–99.37% (Supplementary Table 7). This suggested that the HERA assembled sequences were very similar to the other regions in their sequence quality. These results demonstrated the potential application of HERA to improving the complex regions in human genome assembly.

### De novo assembling Tartary buckwheat Pinku1 genome

Finally, we applied HERA to improve the Tartary buckwheat genome (Table 1). The previously published Tartary buckwheat genome was assembled using only ~30x SMRT sequence data^27^. In this study, we added more SMRT data to a total sequencing depth of ~70×. We used both PBcR and CANU pipelines to generate two different assemblies, which had a contig N50 size of 0.45 Mb and 1.1 Mb, and a total size of 587.7 Mb and 452.1 Mb, respectively (Supplementary Table 2). After HERA assembling based on the PBcR-assembled contigs, the contigs N50 size was increased to 22.24 Mb, with a total genome size of 453.4 Mb, very close to the assembled size of CANU. The hybrid scaffolding with BioNano genome maps resulted in only 14 scaffolds for all chromosomes with five chromosomes in single scaffold. Further gap filling with HERA in the hybrid scaffolds resulted in a total of 20 contigs on eight chromosomes in the final assembly Pink1_HERA1, with a contig N50 size of 27.85 Mb and a single-contig chromosome 8. The assembly of the highly contiguous Tartary buckwheat genome clearly demonstrated the power of HERA in generating chromosome-scale contigs in the assembly of complex plant genomes.

### The running time of HERA

The running time of HERA can be divided into three parts: finding sequence overlaps, building the overlap graph and running the graph traversals. The graph construction and traversals take O(*V* *+* *E*) time due to limited number of paths constructed starting from each anchoring node, and bounded path length between anchoring nodes. For finding sequence overlaps, we primarily used BWA^23^. The running of HERA for assembling the four genomes above was performed on Lenovo ThinkSystem SD530 with Xeon Gold 6130 CPU (2.1 GHz). The total CPU core hours used for the genomes were listed in Table 2. The results showed that the running time of HERA was mainly spent on finding overlaps with BWA (consuming 88.59–96.02% of the total time). Therefore, one of the major ways of improving the speed of HERA is to use a more efficient sequence aligner than the current BWA.

**Table 2:** The running time of HERA.

Genome	Overlap (BWA) hour	Total hour	BWA ratio
R498	2515	2641	95.23%
Pinku1	2922	3043	96.02%
B73	14,409	16,265	88.59%
HX1	17,657	20,141	87.67%

## Discussion

We have reported a highly efficient assembly method, HERA, to resolve repetitive sequences, which is the central objective for all genome assemblers. We demonstrated that HERA could dramatically improve the contiguity and completeness of genome assembly by assembling the previously unassembled repeats including many tandemly repetitive sequences. HERA can generate super-long contigs using SMS data only, and enables the assembly of chromosome-scale contigs by further integrating BioNano genome maps and Hi-C data for filling gaps and resolving repeats. Compared with the simple usage of genome maps and Hi-C data^28^, the benefits of using HERA in genome assembly are multifold: as contig lengths increase, it is more likely for a contig being aligned to genome maps as well as being anchored or positioned or oriented correctly onto chromosomes, and thus the constructed chromosomal sequences contain fewer gaps as well as fewer errors.

The key difference in dealing with repeats between HERA and SG assemblers such as CANU and FALCON is that HERA assembles a repeat by identifying and scoring the sequence as a whole with aim to separate different repeat copies from each other, while the SG assemblers compress all similar reads above a threshold into one, which makes many different repeat copies unresolvable. In the absence of sequence errors, HERA and SG will likely generate the same results. However, in the presence of sequence errors which are unavoidable under the current sequencing technologies, the transitive reduction in SG often generates branching nodes from repeat compression, and thus fragments a repeat into many small contigs. HERA is superior in that an overlap graph is transformed to a connection graph in which the repeats are not compressed. HERA is relatively insensitive to unevenly distributed sequencing or self-correction errors. Based on its function, HERA is naturally complemented to current long-read assemblers so that direct integration of the HERA method is highly recommended. The way an assembler deals with repeats can affect its speed and memory usage, and thus various tradeoffs are often implemented to balance the contig length and program efficiency^1,11,15,29^. By using the HERA method we described here as the second stage in an assembler, the assembly of unique sequences can use a conservative approach by assembling only the high-confidence regions in the first stage. We expect that this strategy will decrease the complexity of the program implementation and increase the speed of new assemblers.

The major limitation of HERA is inherent to all assemblers that when two repeat regions are highly similar (e.g., >99.5% in sequence identity), the current implementation of HERA does not try to resolve the repeat aggressively by default due to potential sequencing errors (the current sequence accuracy is 98.5–99% after self-correction of CANU). HERA can distinguish two segmentally duplicated repeats based on small percentage of sequence variations (<1–2%) by selecting the best paths to connect the pairs of true adjacent flanking sequences. For this reason, the HERA method and the SDA method^22^ are complementary to each other in that the long contigs/paths generated by HERA (not yet connected to their flanking sequences) can be used by SDA as reference sequences to retrieve the reads for further identification of paralogous sequence variants. In the future, this limitation may be removed by using the CCS reads from PacBio, which has sequence accuracy >99%^30^. HERA’s performance is also dependent of the sequence aligners (the sequence overlappers in assemblers). We primarily used BWA^23^ to do sequence alignment for our assembled genomes, but we found that some overlaps between reads could be missed. Therefore, an optimized sequence aligner will be helpful for improving the performance of HERA.

For filling known gaps detected by genome maps or present in existing genomes, HERA is a preferred method compared to the existing gap-fillers since HERA performs local genome assembly instead of simple sequence extension. We filled 96.9% of the gaps (only 76 left) of B73 and anchored additional sequences and genes onto chromosomes. We also showed that more than 80% of the InDels detected by BioNano genome maps in PBcR-assembled B73 sequences could be fixed by HERA. Comparisons between the HERA assembly of HX1 and the human GRCh38 reference genome showed that many gaps in GRCh38 could also be filled, and that GRCh38 contained some potential errors that could be fixed. These results suggest that HERA can serve as a local genome assembly method or curation tool to improve the contiguity and completeness of complex genomes, including the correction of existing assembly errors.

## Methods

### The problem description for HERA algorithm

Given a set of known DNA sequences (KDSs) of unknown genome location and a set of WGS long sequencing reads, with two KDSs being called adjacent if there are no other KDSs being present between them on chromosome, our objective is to connect as many adjacent KDSs as possible by assembling the missing regions between them using the reads without introducing chimeric error, which occurs when two non-adjacent KDSs on genome are connected by an assembled sequence.

### Input data types and definitions in HERA

HERA uses self-corrected WGS SMS reads and pre-assembled contigs as input data. The contigs can be generated by any of existing assemblers such as PBcR, CANU, MECAT, or FALCON. The raw SMS reads were corrected with the self-correction module of CANU. Obviously, each contig has an assumed genome location even though it is unknown. Here we call the pre-assembled contigs anchoring sequences (A-seqs) as they delimit the genomic regions to be assembled with read sequences (R-seqs). Two contigs are called adjacent if there are no other contigs being present between them on chromosome.

All A-seqs and R-seqs are compared to each other to identify their sequence overlaps. A piece of DNA sequence, either an A-seq or an R-seq, has two ends. Given *L*_se_, a sequence end is defined to be a sequence that is up to *L*_se_ kb from the end. A sequence can be extended at one end by another sequence if they are overlapping and the latter is not entirely covered by the former, i.e., the overlap is shorter than the latter. A sequence end can be extended iteratively to form a tiling path of overlapping sequences.

Given a pair of overlapping sequences S1 and S2 that are not entirely covered in either way, each contains an overlap region with lengths OL1 and OL2, sequence identity *SI*, overhang lengths OH1 and OH2, and extension lengths EL1 and EL2 (Supplementary Fig. 1a). We define the following scores: the overlap score of S1 and S2 OS *=* (OL1 + OL2)*SI/2, the extension score of S2 extending S1 ES2 = OS + EL2/2 - (OH1 + OH2)/2, and similarly for the extension score of S1 extending S2. For a tiling path containing more than one overlaps, the score (either overlap or extension) of a tiling path is the average of all overlaps in the path. The extension score between a pair of A-seq and R-seq is always the score of the R-seq extending the A-seq.

Due to sequencing errors or variations between repeat copies, the pair of sequences in an overlap may not be the same. We therefore define a global minimum sequence identity cutoff parameter SI_min_ for filtering out low-confidence overlaps. The average base accuracy (*α*) of all reads is estimated using the average sequence identity of all high-confidence overlaps by allowing the number of overlapping sequences for each sequence to be at most the average sequence depth. SI_min_ is typically set to a value smaller than but close to *α*. The average sequencing error rate in all reads is *ε* *=* *1-α*.

### Overlap graph construction in HERA

HERA implements an undirected overlap graph, *G*_o_(*V*, *E*), with *V* representing sequences and *E* representing sequence overlaps. The set *V* (*V* = {*A*,*R*}) consists of two types of nodes: anchoring nodes (*A* = { *a*_*0*_,…, *a*_*i*_ }) for A-seqs, and read nodes (*R* = { *r*_*0*_,…, *r*_*j*_ }) for R-seqs. Note that since each node represents a sequence, a node must store the sequence ends (*d* for head *h* or tail *t*) explicitly inside (i.e., each node has a direction). An edge from *E* represents the overlap between two sequences. The edges incident on a node are divided into two groups, one on each end of the node. For simplicity, we do not always explicitly indicate the node ends if it is clear in the text. Each read node end is allowed to connect to at most one anchoring node with the highest overlap score (randomly selecting one for equal scores). In *G*_o_, the connection of two anchoring nodes can be found by traversing the graph using depth-first-search to generate traversal paths (or simply paths) that represent sequence tiling paths. To generate a valid tiling path, a traversal of the graph must obey the following rule: whenever it goes into a node from one end *a*^*h*^, it must exit from the other end *a*^*t*^, and vice versa.

We denote two anchoring nodes adjacent if they represent two adjacent contigs. We denote two anchoring nodes ‘directly connected’ if they are connected through a path in *G*_o_ not including any other anchoring nodes. A path *p*_*ijx*_ = {*a*_*i*_^*d*^, *r*_*ix0*_, …, *r*_*ixk*_, *a*_*j*_^*d*^} between two adjacent anchoring nodes (AANs) (*a*_*i*_, *a*_*j*_) represents an assembled contig that connects them. The score of a path *p* in *G*_o_ is the same as the score of the tiling path represented by *p*. Therefore, the key issue in the assembly problem described above is to identify the largest possible number of AAN pairs in *G*_o_, for which a heuristic algorithm is described as follows.

### Finding paths between anchoring nodes in HERA

An anchoring node, *a*_*i*_ ∈ *A* can be directly connected to more than one anchoring nodes, *A*_*i*_ = {*a*_*i0*_,…*, a*_*ij*_} ⊂ *A*, from which we try to identify the AAN of *a*_*i*_. There are generally multiple paths from *a*_*i*_ to each *a*_*ij*_ (*P*_*ij*_ = {*p*_*ij0*_, …, *p*_*ijx*_}). Intuitively, the overlaps between the reads originating from the same repeat copy should generally have larger score than those between the reads originating from different repeat copies. Therefore, the path scores between the AANs should be higher than those between non-AANs. Hence, a naïve method is to select the highest-scored path among all paths *P*_*i*_ = {*P*_*i0*_, …, *P*_*ij*_} from *a*_*i*_ to every *a*_*ij*_ and choose the ending anchoring node on the best-scored path as the AAN of *a*_*i*_. However, this can easily lead to incorrect selections due to sequencing errors. On the other hand, the traversal will not likely pass from the reads of one repeat copy to those of another unless the sequence similarity between the two repeat copies is higher than the pre-defined threshold. Since the sequencing errors occur randomly, we can reasonably assume that the number of paths between a pair of AANs is generally higher than that between two non-AANs. Therefore, a better way is to use the high-scoring path number (instead of only path score) for identifying the AAN of *a*_*i*_.

It is computationally expensive to enumerate all allowed paths emanating from *a*_*i*_ during graph traversal, and thus we limit the paths chosen for the algorithm. We only consider paths representing sequences whose length is at most a predefined value, denoted as *L*_me_. We also require that each read node (or read for simplicity) can be used only once in constructing a path. A path extension always stops when reaching at a read node that connects to another anchoring node which defines the path end. To control the computational time and maximize the chance to find a correct path in the presence of sequencing errors, we utilize a combination of fixed scoring schemes and a graph random walk approach to construct a set of high-scoring paths _*Pi*_ that directly connect *a*_*i*_ to other anchoring nodes.

Approach I. In the first extension step, all reads connecting to *a*_*i*_ are selected to extend *a*_*i*_. For further extension, only the read with the highest overlap score is selected. If scores for two reads are tied, the read with higher sequence identity or the longer read is selected or randomly selected for the rest. Note that not all paths generated in the first step can eventually reach to another anchoring node. In the case of dead end where no more connected read nodes can be found, the extension step goes back to the previous node, and then is extended by the next top-scored read. This step generates a set of paths *P*_*i*_^1^.

Approach II. Similar to approach I, except that in each extension step, a read with the highest extension score is selected. This step generates a set of paths *P*_*i*_^2^.

Approach III. A random walk approach is used to randomly select a connected read for each extension. The probability of a connected read being selected is proportional to its extension score. When a path extension stops no matter whether an anchoring node is reached, the trial for constructing a path is finished, and a new trial can start over. For each starting anchoring node *a*_*i*_, a limited number of trials may be performed to control the running time. This step generates a set of paths *P*_*i*_^3^.

Approaches I and II generate a fixed number of paths while the approach III can generate arbitrary number of paths. By default we require that the approach III generates more paths than approaches I and II. All the paths are put together and duplicated paths are removed to obtain a non-redundant set of paths *P*_*ij*_ ⊂ *P*_*i*_ = {*P*_*i*_^1^, *P*_*i*_^2^, *P*_*i*_^3^} = { *P*_*i0*_, …, *P*_*ij*_ } from *a*_*i*_ to each anchoring nodes *a*_*ij*_.

### Generation of consensus sequences in HERA

Among all paths *P*_*ij*_ between a pair of anchoring nodes, we need to find a representative or consensus sequence *s*_*ij*_ as a candidate assembled sequence. Note that not all these paths have the same length due to sequence errors and variations between different repeat copies. If the path lengths are distributed within a small range, such as <10 kb, then all the paths are put into one group. Otherwise, all sequences were sorted according to their lengths from short to long and divided into 1-kb windows. Comparing each window with the ones immediately before and after it, the window with the largest sum of the path frequencies is designated as a peak window, and the window with the smallest sum of the path frequencies as a valley window. If the lowest path length frequency in a valley window is less than a predefined proportion, such as 90%, of the highest path length frequency in its right peak window, then the path length of the lowest frequency in the valley windows (left-inclusive) is used to divide the whole set of paths to separate groups, and finally we have *P*_*ij*_ = { *P*_*ij0*_, …, *P*_*ijg*_ }.

In each group *P*_*ijg*_, all paths with length frequency less than half of the highest path length frequency are discarded. The sequences of the remaining paths are aligned to each other. Only the alignments between a pair of sequences with minimum mutual coverage of 95% are considered to be valid. Finally, one of the paths with the highest length frequency matching to the largest number of other paths is selected as the consensus sequence *s*_*ijg*_. The number of paths *n*_*ijg*_ matching to the consensus sequence *s*_*ijg*_ is designated as the high-scoring path number in this group.

When multiple groups of paths are found between a pair of anchoring nodes, a consensus sequence will be generated for each group. The multiple consensus sequences *S*_*ij*_ = { *s*_*ij0*_, …, *s*_*ijg*_ } of different length indicate the presence of multiple similar repeat units. If the length of the region to be assembled is known (e.g. for gap filling), the consensus sequence corresponding to the known length was selected. Otherwise, starting from the group with the highest path length frequency, its high-scoring path number is compared with that of next immediate group with longer paths, iteratively. If there are only two groups or the high-scoring path number in the longer path group is more than half of that in the shorter path group, then the longer path group will be selected. Otherwise, the shorter path group will be selected. Finally the selected group of paths *P*_*ijk*_ with consensus sequence *s*_*ijk*_ is used as the paths between the pair of anchoring nodes.

If the paths in the set *P*_*ijk*_ have a wide length distribution (>100 kb) and cannot be divided into separate groups, the repeat underlying the paths is viewed as a complex repeat. By default, these kinds of repeats are not used to connect the anchoring sequences, i.e., the region is not assembled.

### Construction of connection graph in HERA

After a consensus sequence is determined between every pair of connected anchoring nodes, a connection graph *G*_c_(*V’*, *E’*) (Fig. 1d) is constructed, consisting of only the anchoring nodes of *G*_o_ (*V’* = *A*), and the consensus sequences of the paths between each pair of anchoring nodes as edges. The edge length is the sum of high-scoring path numbers (NP) on both directions between each pair of anchoring nodes.

To identify segmentally duplicated sequences with high sequence similarity, we define a conflict index (CI) for each end of each anchoring node. For any anchoring node end *a*_*i*_^*d*^ with connected anchoring nodes *A*_*ij*_, by selecting its longest incident edge length NP_*im*_ and second longest edge length NP_*in*_, we define the conflict index for *a*_*i*_^*d*^ as CI_*i*_^*d*^ = NP_*in*_/NP_*im*_. We denote that an anchoring node end has conflicting connections if its conflict index is larger than a predefined threshold, CI_max_.

### Final sequence assembly in HERA

For an anchoring node end without conflicting connections, its AAN is the node connected with its longest incident edge on the connection graph. The anchoring node ends with conflicting connections are not connected to the consensus sequence obtained above unless the conflict is resolved. The final sequence assembly is straightforward: the non-conflicting anchoring node ends are connected to their AANs using the consensus sequences between them to generate super-contigs.

### SMRT sequencing

The leaf DNA of Tartary buckwheat cultivar Pinku1 was used to prepare SMRTbell libraries (20 kb inserts) with the standard protocol provided by PacBio (Pacific Biosciences, USA), and the sequencing was conducted on a PacBio Sequel system. Sequence reads with a quality score below 0.8 were discarded.

### Assembly of rice, maize, human, and Tartary buckwheat genomes

The raw SMRT reads of R498, Tartary buckwheat, B73, and HX1 were corrected using the CANU pipeline with default parameters. The corrected reads of R498 were further assembled into sequence contigs using CANU with default parameters. The CANU assembled R498 contigs and the published contigs in B73 RefGen_v4 and HX1 (HX1_FALCON) were used for HERA assembly with the corrected SMRT reads. The raw reads of Pinku1 were assembled using both the CANU and PBcR pipeline with default parameters and the assemblies were improved with HERA. For each genome, the contigs of at least 50 kb were used as anchoring contigs to construct overlap graphs with the corrected reads and the contigs <50 kb. All contigs and the corrected reads were aligned all-against-all with Minimap2 (https://github.com/lh3/minimap2) and BWA^23^ with default parameters to identify the sequence overlaps. To reduce memory usage and computational cost, a reduced overlap graph was constructed. The reads that are aligned to the middle of the contigs with both coverage and identity >99.5% or fully contained by other reads were discarded and overlaps with sequence similarity <97% were also discarded (SI_min_ set to 97%). The newly assembled Pinku1 scaffolds were clustered onto chromosomes to form pseudomolecules using 3D-DNA software^31^.

The genomes were assembled using HERA with *L*_se_ set to 25 kb, *L*_me_ set to 800 kb, and CI_max_ set to 0.75. The HERA assembled super-contigs were combined with BioNano genome maps to generate hybrid maps, and HERA was used again to fill in the gaps in the hybrid maps. The resulting contigs were further connected with HERA and validated with genome maps. The contigs were mapped to reference genomes by BWA^23^ for clustering except those in Pinku1 which were clustered using 3D-DNA. The order and group information of the contigs based on reference genomes were used to resolve conflicts during the process of HERA assembly, but it is not used for determining gaps between contigs. This generated super-contigs. For simplicity, the final order of the HERA assembled super-contigs on chromosomes was determined based on their alignment to reference genome. Except the R498 genome, no further gap filling was performed after the super-contigs were anchored onto chromosomes.

### Sequence alignments to genomes

The BAC sequences and human genomes downloaded from GenBank nucleotide database, Illumina short reads, and the corrected SMRT reads were aligned to the assembled genome using BWA-mem (http://bio-bwa.sourceforge.net/) default setting. After alignment, only one of the best matched alignments for each read was selected to compute the sequence identity and genome coverage.

### Cleaning of the Tartary buckwheat assembly

The HERA assembled Tartary buckwheat contigs were aligned to cpDNA and mtDNA, microbial and human DNAs with BWA-mem and the contigs with alignment length >500 bp were discarded. The remaining contigs between 20–50 kb were aligned to the longer ones and those with sequence coverage >90% were also discarded.

### BioNano map assembly and gap filling

The de novo and hybrid assembly of the BioNano genome maps were performed using the IrysView software (BioNano Genomics). A minimum length of 150 kb was used as a cutoff in de novo assembly. To fill a gap with known length in hybrid scaffolds, HERA selected a path whose length was the closest to the gap length and whose enzyme nicking sites matching the genome map.

### Reporting summary

Further information on research design is available in the Nature Research Reporting Summary linked to this article.

## Supplementary information

Reporting Summary

Supplementary Information

## Data Availability

Rice data (R498) were previously published^5^ with the sequence reads available at GSA database (http://gsa.big.ac.cn/index.jsp) under project PRJCA000313. The maize data were downloaded from NCBI under BioProject number PRJNA10769 and SRA accessions SRX1472849. The human data were downloaded from http://hx1.wglab.org/ and NCBI under BioProject PRJNA301527. The sequence reads of Tartary buckwheat was deposited into GSA database under project PRJCA000402. The HERA assembled genome sequences of B73, HX1, and Pinku1 were deposited into BIG Data Center (http://bigd.big.ac.cn/gwh) under accession numbers GWHAAEN00000000, GWHAAEM00000000 and GWHAAEO00000000. The HERA assembled genome sequences are also available at http://mbkbase.org/R498, /http://mbkbase.org/B73, http://mbkbase.org/HX1 and http://mbkbase.org/Pinku1.

## Code availability

The HERA software is coded in Perl and can be freely downloaded at https://github.com/liangclab/HERA. HERA is free for academic and research use.
