# Supplementary material for: Assembly of chromosome-scale contigs by efficiently resolving repetitive sequences with long reads
Source: Nat Commun. 2019 Nov 25;10:5360. doi: 10.1038/s41467-019-13355-3 (PMC6877557; doi:10.1038/s41467-019-13355-3)
Supplement: Supplementary file 1 — Reporting Summary [file 41467_2019_13355_MOESM1_ESM.pdf]

nature research

Corresponding author(s): Chengzhi Liang

Last updated by author(s): Oct 7, 2019

Reporting Summary

Nature Research wishes to improve the reproducibility of the work that we publish. This form provides structure for consistency and transparency in reporting. For further information on Nature Research policies, see Authors & Referees and the Editorial Policy Checklist .

Statistics

For all statistical analyses, confirm that the following items are present in the figure legend, table legend, main text, or Methods section.

n/a	Confirmed	
☒	☐	The exact sample size (*n*) for each experimental group/condition, given as a discrete number and unit of measurement
☒	☐	A statement on whether measurements were taken from distinct samples or whether the same sample was measured repeatedly
☒	☐	The statistical test(s) used AND whether they are one- or two-sided<br>*Only common tests should be described solely by name; describe more complex techniques in the Methods section.*
☒	☐	A description of all covariates tested
☒	☐	A description of any assumptions or corrections, such as tests of normality and adjustment for multiple comparisons
☒	☐	A full description of the statistical parameters including central tendency (e.g. means) or other basic estimates (e.g. regression coefficient) AND variation (e.g. standard deviation) or associated estimates of uncertainty (e.g. confidence intervals)
☒	☐	For null hypothesis testing, the test statistic (e.g. *F*, *t*, *r*) with confidence intervals, effect sizes, degrees of freedom and *P* value noted<br>*Give P values as exact values whenever suitable.*
☒	☐	For Bayesian analysis, information on the choice of priors and Markov chain Monte Carlo settings
☒	☐	For hierarchical and complex designs, identification of the appropriate level for tests and full reporting of outcomes
☒	☐	Estimates of effect sizes (e.g. Cohen's *d*, Pearson's *r*), indicating how they were calculated

Our web collection on statistics for biologists contains articles on many of the points above.

Software and code

Policy information about availability of computer code

Data collection	All data were collected by downloading from NCBI with the tool of "wget"
Data analysis	CANU (canu-1.5), Falcon(fc_env_180419) and PBcR(wgs-8.3rc2) assemblers<br>BWA : version 0.7.10<br>The software HERA (Version 1) was included with the manuscript. The software HERA can be used as instructed in readme.txt.

For manuscripts utilizing custom algorithms or software that are central to the research but not yet described in published literature, software must be made available to editors/reviewers. We strongly encourage code deposition in a community repository (e.g. GitHub). See the Nature Research guidelines for submitting code & software for further information.

Data

Policy information about availability of data

All manuscripts must include a data availability statement. This statement should provide the following information, where applicable:

- Accession codes, unique identifiers, or web links for publicly available datasets
- A list of figures that have associated raw data
- A description of any restrictions on data availability

Rice data (R498) was previously published with the sequence reads available at GSA database (http://gsa.big.ac.cn/index.jsp) under project PRJCA000313. The maize data was downloaded from NCBI under BioProject number PRJNA10769 and SRA accessions SRX1472849. The human data was downloaded from http://hx1.wglab.org/ and NCBI under BioProject PRJNA301527. The sequence reads of Tartary buckwheat was deposited into GSA database under project PRJCA000402. The HERA assembled genome sequences of B73, HX1, and Pinku1 were deposited into BIG Data Center (http://bigd.big.ac.cn/gwh) under accession numbers GWHAAEN00000000, GWHAAEM00000000 and GWHAAEO00000000. The HERA assembled genome sequences are also available at http://mbkbase.org/B73/, http://mbkbase.org/HX1/ and http://mbkbase.org/Pinku1/

Field-specific reporting

Please select the one below that is the best fit for your research. If you are not sure, read the appropriate sections before making your selection.

☒ Life sciences ☐ Behavioural & social sciences ☐ Ecological, evolutionary & environmental sciences

For a reference copy of the document with all sections, see nature.com/documents/nr-reporting-summary-flat.pdf

Life sciences study design

All studies must disclose on these points even when the disclosure is negative.

Sample size	No statistical method was used to calculate the sample size. We randomly selected four genomes to test our software HERA, and these four genomes are rice R498, human HX1, maize B73 and Tartary buckwheat Pinku1.
Data exclusions	The raw pacbio long reads with the length of less than 1 Kb were excluded.<br>Corrected long reads with the length of less than 5 Kb were excluded.
Replication	The software HERA was tested on many genomes and each data were tested multiple times.
Randomization	No randomization because all data were used together without group allocation.
Blinding	No blinding because no data group was allocated during data collection and analysis.

Reporting for specific materials, systems and methods

We require information from authors about some types of materials, experimental systems and methods used in many studies. Here, indicate whether each material, system or method listed is relevant to your study. If you are not sure if a list item applies to your research, read the appropriate section before selecting a response.

Materials & experimental systems

n/a	Involved in the study	
☒	☐	Antibodies
☒	☐	Eukaryotic cell lines
☒	☐	Palaeontology
☒	☐	Animals and other organisms
☒	☐	Human research participants
☒	☐	Clinical data

Methods

n/a	Involved in the study	
☒	☐	ChIP-seq
☒	☐	Flow cytometry
☒	☐	MRI-based neuroimaging
