# Supplementary material for: Assembly of chromosome-scale contigs by efficiently resolving repetitive sequences with long reads
Source: Nat Commun. 2019 Nov 25;10:5360. doi: 10.1038/s41467-019-13355-3 (PMC6877557; doi:10.1038/s41467-019-13355-3)
Supplement: Supplementary file 2 — Supplementary Information [file 41467_2019_13355_MOESM2_ESM.pdf]

SUPPLEMENTARY INFORMATION

Assembly of chromosome-scale contigs by efficiently resolving repetitive sequences with long reads

Huilong Du and Chengzhi Liang

Supplementary Figures

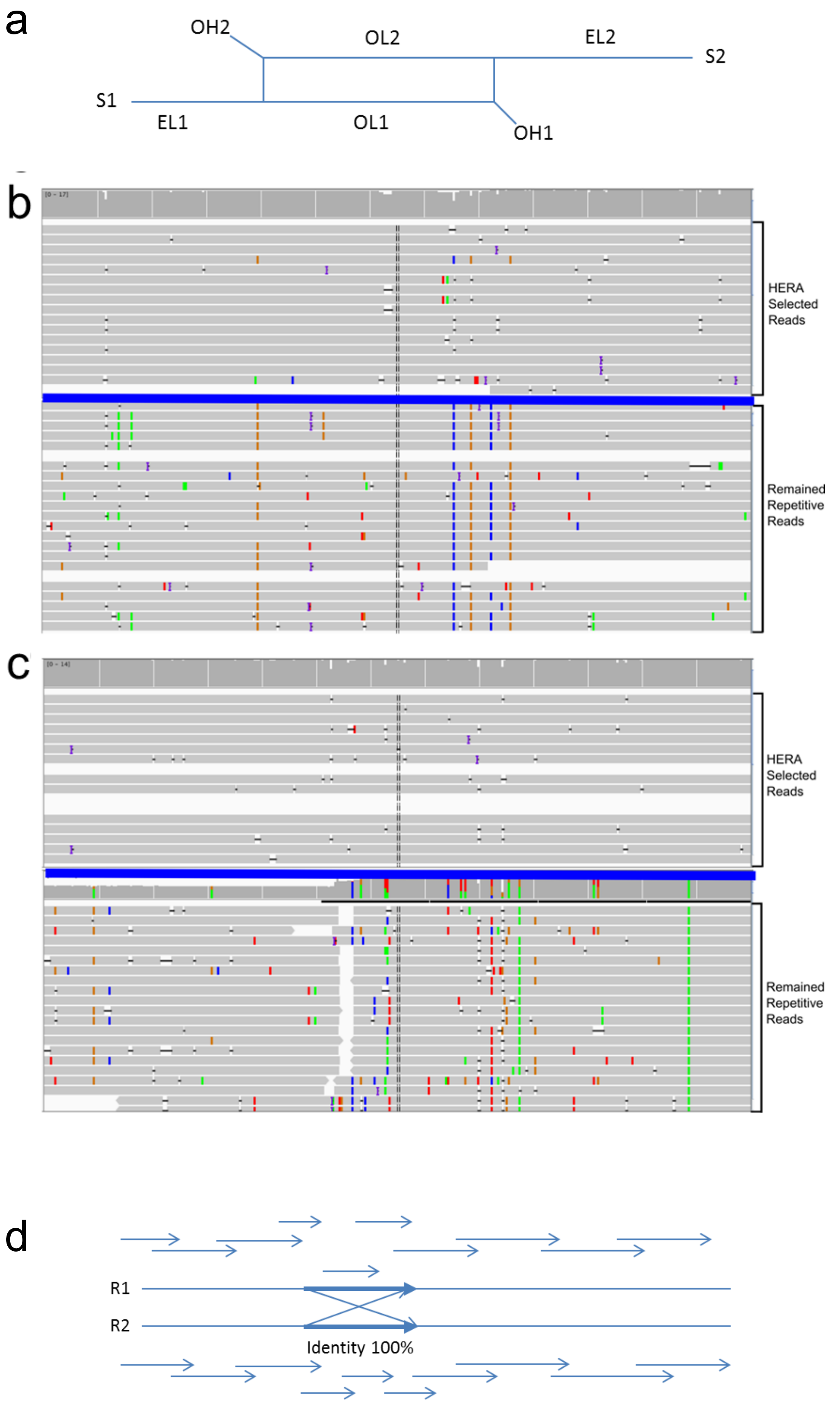

Supplementary Figure 1: Diagrams showing sequence overlaps and read tiling paths and comparison of selected reads with non-selected reads by HERA in a repeat region.

(**a**) Two sequences with an overlap. OL, overlap length; OH, overhang length; EL, extension length. (**b**)(**c**) Two repetitive sequences of ~23 kb on R498 chromosome 2 assembled by HERA shared sequence identity of 97.83% that can be distinguished at read level due to sequence variations. The colored bars from IGV (http://software.broadinstitute.org/software/igv/) screenshot indicated SNPs between the HERA assembled sequences and the reads. Notably the reads can be classified into two groups that can be distinguished from each other based on the SNP distribution: the reads selected by HERA are originated from the same copy (upper panel) and the reads not selected by HERA are originated from the other repeat copy (lower panel). CANU failed to assemble the two repeats and generated several contigs for each region. (**d**) Read tiling paths in two repeat copies R1 and R2. Note that a path selected by HERA can start from reads of R1 and end at reads of R2 and vice versa through the reads in the identical region. Thus the number of paths that can be randomly selected ending at either R1 or R2 will be very similar to each other, which causes conflicts in the connection graph.

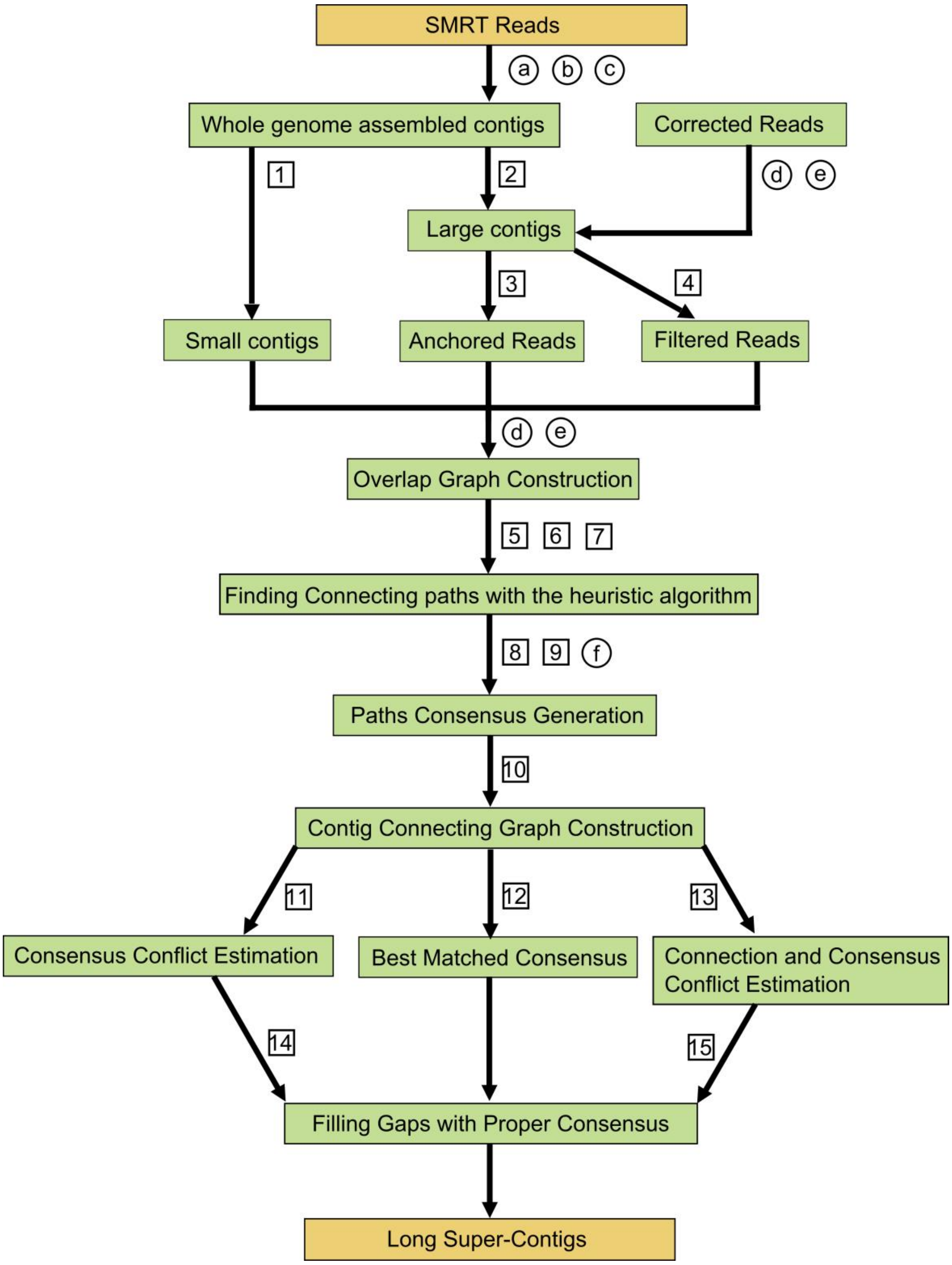

Supplementary Figure 2: Work flow of data process and HERA assembly.

External software: (a) PBcR. (b) CANU. (c) FALCON. (d) MiniMap2. (e) BWA. (f) Daligner.

(1)(2) Selecting sequences by length. (3) Selecting the best matched reads which are aligned to the head or tail of contigs as starting/ending reads. (4) Removing the reads that are completely aligned to the internal region of contigs. (5) Using the highest overlap score for read extension. (6) Using the longest extension length for read extension. (7) A Random Walk approach for read extension. (8)(9)(10) Construction of connection graph. (11) Using the contig grouping information from Hi-C clustering, genetic maps, or reference genomes. (12) Using the gap length information from BioNano genome maps. (13) Not using any additional information. (14) (15) Determining the proper contig pairs based on the conflicting indices and the proper consensus sequences for connection.

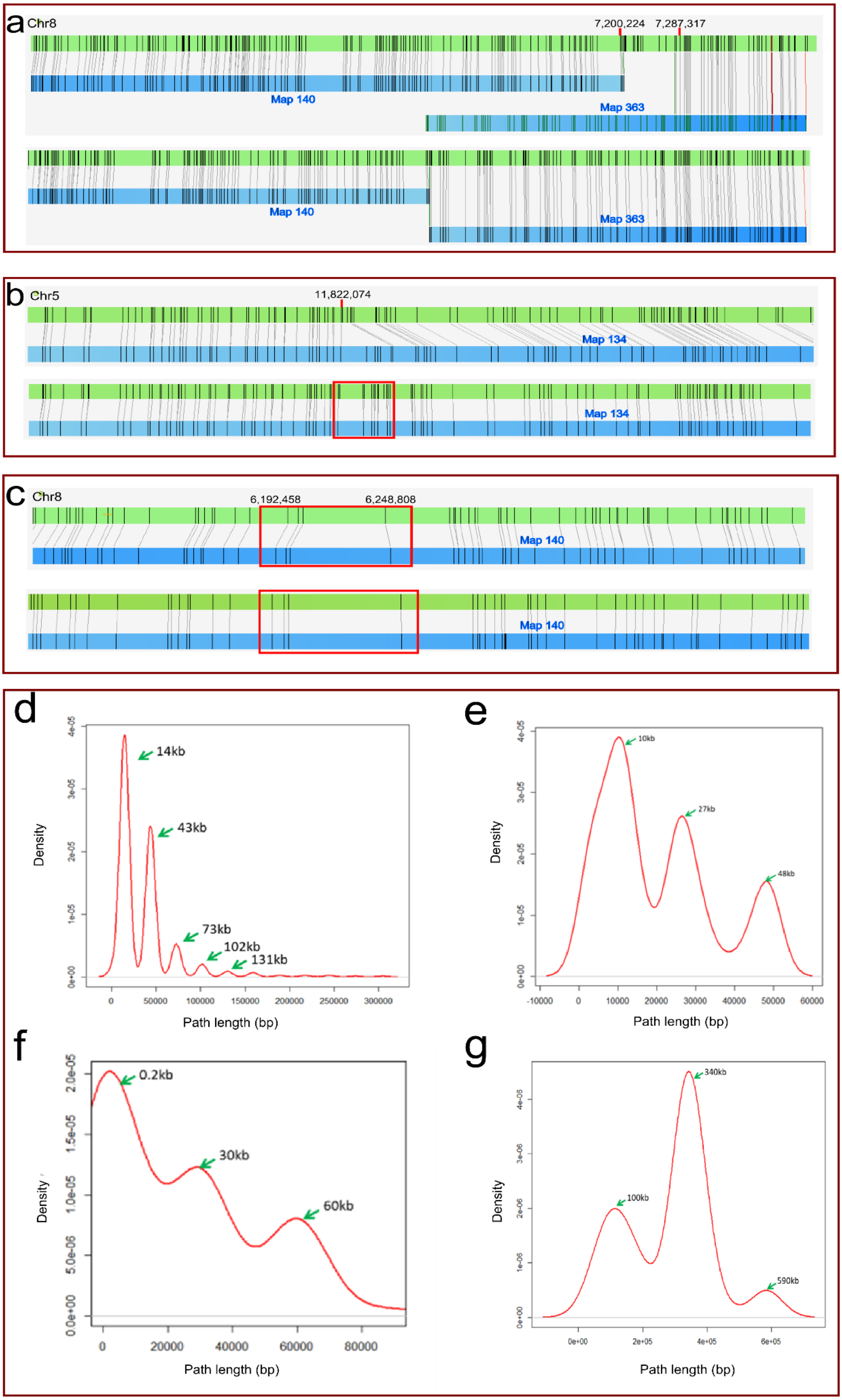

a
Chr8
7,200,224
7,287,317
Map 140
Map 363
Map 140
Map 363
b
Chr5
11,822,074
Map 134
Map 134
c
Chr8
6,192,458
6,248,808
Map 140
Map 140
d
14kb
43kb
73kb
102kb
131kb
Density
Path length (bp)
e
10kb
27kb
48kb
Density
Path length (bp)
f
0.2kb
30kb
60kb
Density
Path length (bp)
g
340kb
100kb
590kb
Density
Path length (bp)

Supplementary Figure 3: The examples of assembling repetitive regions in R498 by HERA.

(**a**) The HERA assembly contained a missing sequence of 387 kb on chromosome 8 in our previously published R498 genome. (**b**) The assembly of a repetitive region by HERA on chromosome 5 around 11,822,074 bp. (**c**) The assembly of a repetitive region by HERA on chromosome 8 around 6,195,389 bp. (**d-g**) Multiple peaks in the connecting path length distribution plots suggested the presence of multiple repeat units. (**d**) Around 7,986,535 bp on chromosome 1. (**e**) Around 16,466,293 bp on chromosome 7. (**f**) Around 6,022,125 bp on chromosome 8. (**g**) Around 14,965,388 bp on chromosome 9.

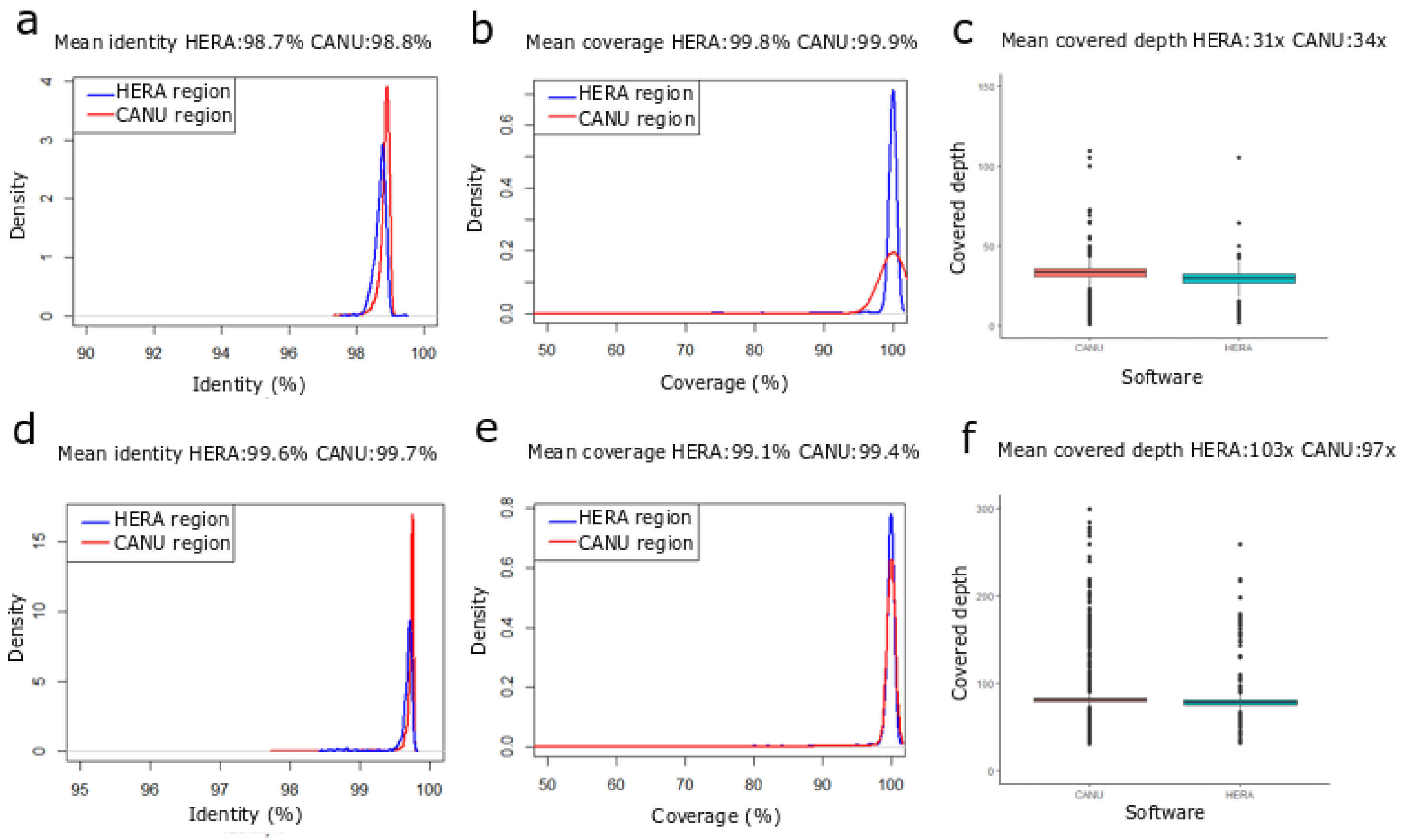

Supplementary Figure 4: Validation of the nucleotide quality of the HERA assembled sequences in R498 genome.

The short reads or the self-corrected long reads were aligned to the whole genome. The best match is selected for each read; for multiple mappings with the same score, one is randomly selected. The HERA assembled regions are compared to CANU assembled regions for (**a,d**) sequence identity, (**b,e**) sequence coverage, and (**c,f**) sequencing depth. (**a-c**) Long reads. Sequencing depth of the whole genome on average: ~34x. (**d-f**) Short reads. Sequencing depth of whole genome: ~97x.

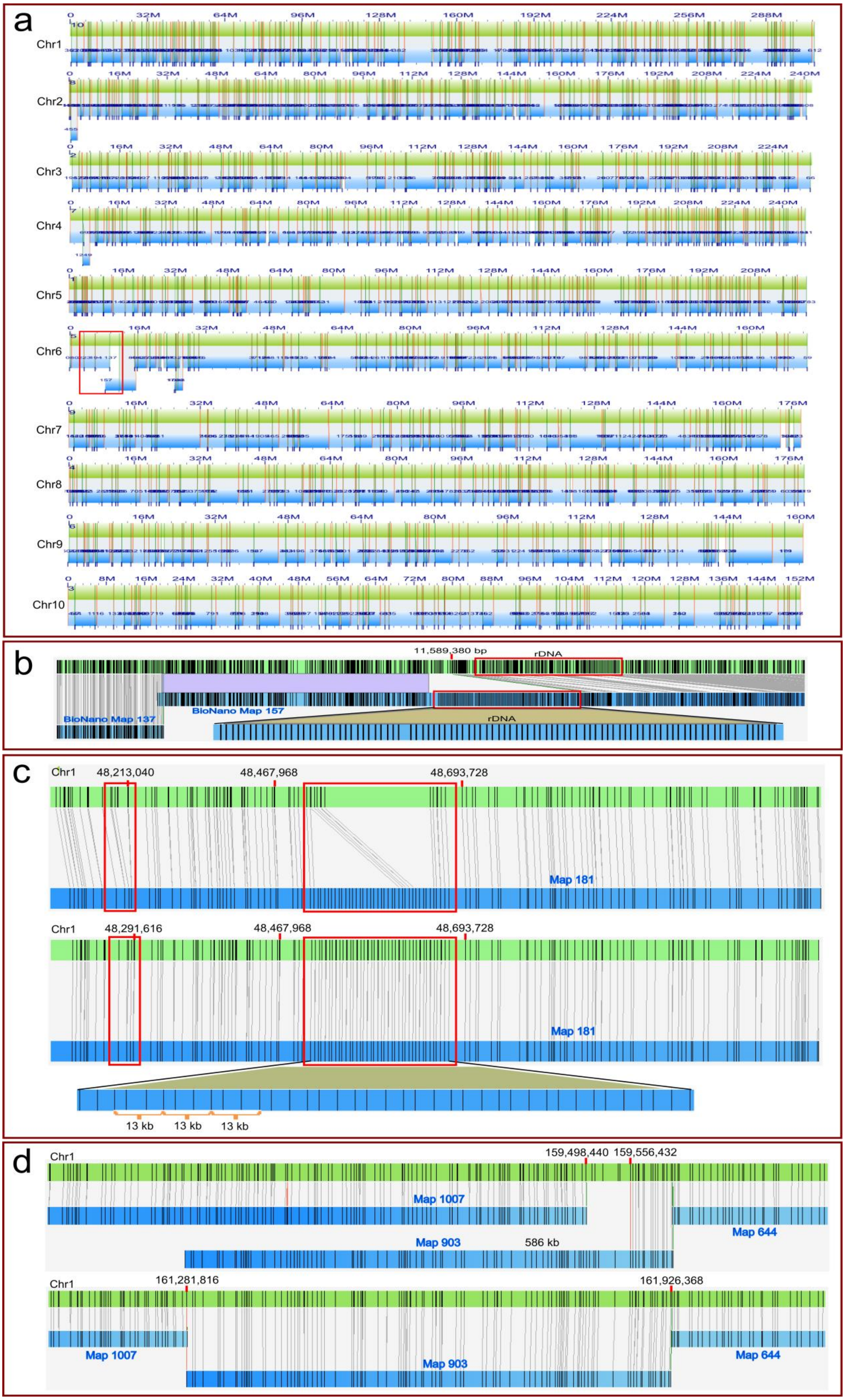

a
Chr1
Chr2
Chr3
Chr4
Chr5
Chr6
Chr7
Chr8
Chr9
Chr10
b
11,589,380 bp
rDNA
BioNano Map 137
BioNano Map 157
rDNA
c
Chr1
48,213,040
48,467,968
48,693,728
Map 181
Chr1
48,291,616
48,467,968
48,693,728
Map 181
13 kb
13 kb
13 kb
d
Chr1
159,498,440
159,556,432
Map 1007
Map 903
586 kb
Map 644
Chr1
161,281,816
161,926,368
Map 1007
Map 903
Map 644

Supplementary Figure 5: Comparison of HERA constructed maize genome to BioNano maps. (**a**) Comparison of HERA assembly to BioNano maps showed nearly perfect alignment with only one region of visible mismatch (red box). (**b**) The mismatch shown in (a) was actually an artifact that was caused by a deletion on the right side of the mismatch. The deletion in the HERA contig is caused by a long stretch of tandem repeats of rDNAs. (**c**) A missing sequence of ~150 kb on chromosome 1 of B73 RefGen_v4 is a tandem repeat with the unit length of 13 kb (right red box), which were correctly assembled by HERA. A small contig was misplaced in RefGen_v4 (left red box), which was corrected in the HERA assembly. (**d**) A missing sequence of ~586 kb on chromosome 1 of B73 RefGen_v4 was assembled correctly by HERA.

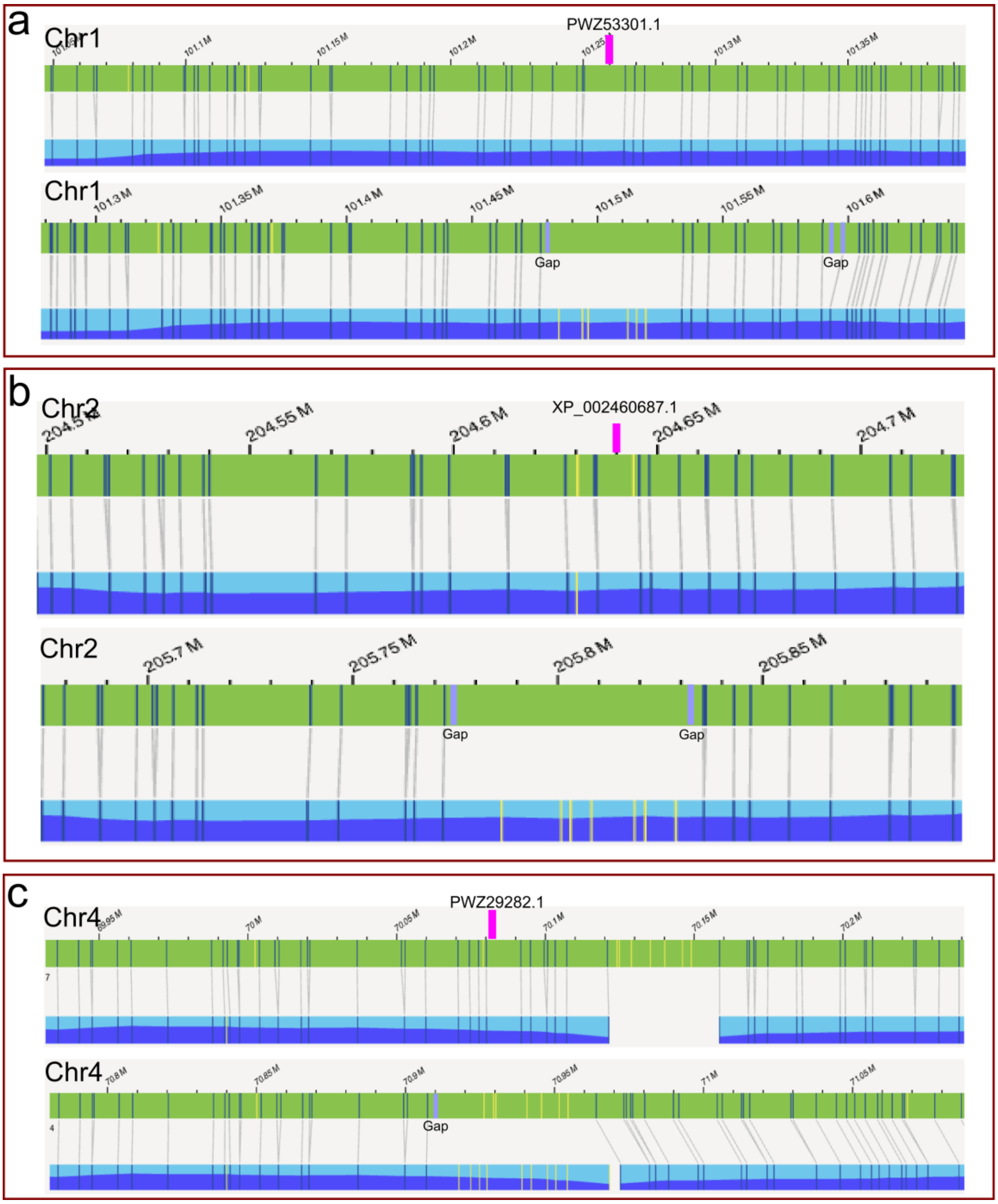

Supplementary Figure 6: Three important maize genes that were found in HERA assembled B73 sequence but not present in RefGen_v4.

The protein sequences of maize genes (their homolog in soghum in parenthesis) (**a**) PWZ53301.1 (XP_002464891.1), (**b**) ACG34567.1 (XP_002460687.1), and (**c**) PWZ29282.1 (XP_002444142.1) were aligned to both B73_HERA1 (upper panel) and RefGen_v4 (lower panel) to examine the presence/absence of the genes. Each of the regions including the genes was correctly assembled in B73_HERA1 but was missing in RefGen_v4.

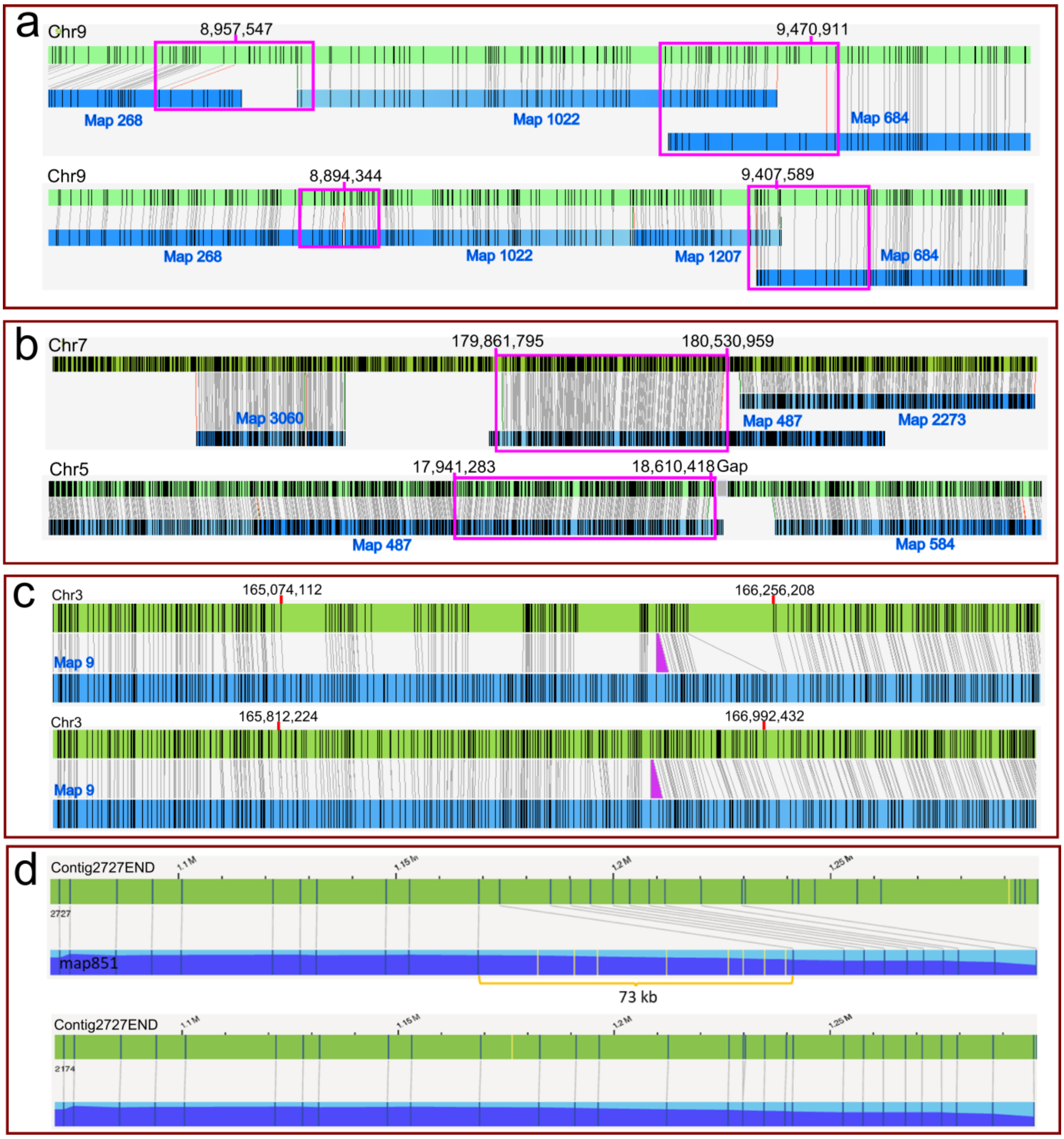

Supplementary Figure 7: The examples of missing sequences in B73 RefGen_v4 that were corrected by HERA.

(**a**) A region with misoriented and missing sequences on maize chromosome 9 in RefGen_v4. In the upper panel, two overhangs (63 kb and 360 kb) due to missing sequences were shown in Map 268 and Map 684 (red boxes), with a misoriented sequence of 513 kb in the middle (corresponding to Map 1022). The error was fixed in the HERA assembly (lower panel). (**b**) A misplaced sequence in RefGen_v4 caused the BioNano map 487 being partially aligned to two non-adjacent regions in RefGen_v4 (upper panel). The error was fixed in the HERA assembly (lower panel). (**c**) A region with five gaps on chromosome 3 of RefGene_v4 from 165.07 Mb to 166.25 Mb was assembled by HERA. Note that HERA did not try to fix the deletion introduced by PBcR in the purple triangle in (c). (**d**) HERA fixed a large InDel error in the middle of a contig assembled by PBcR.

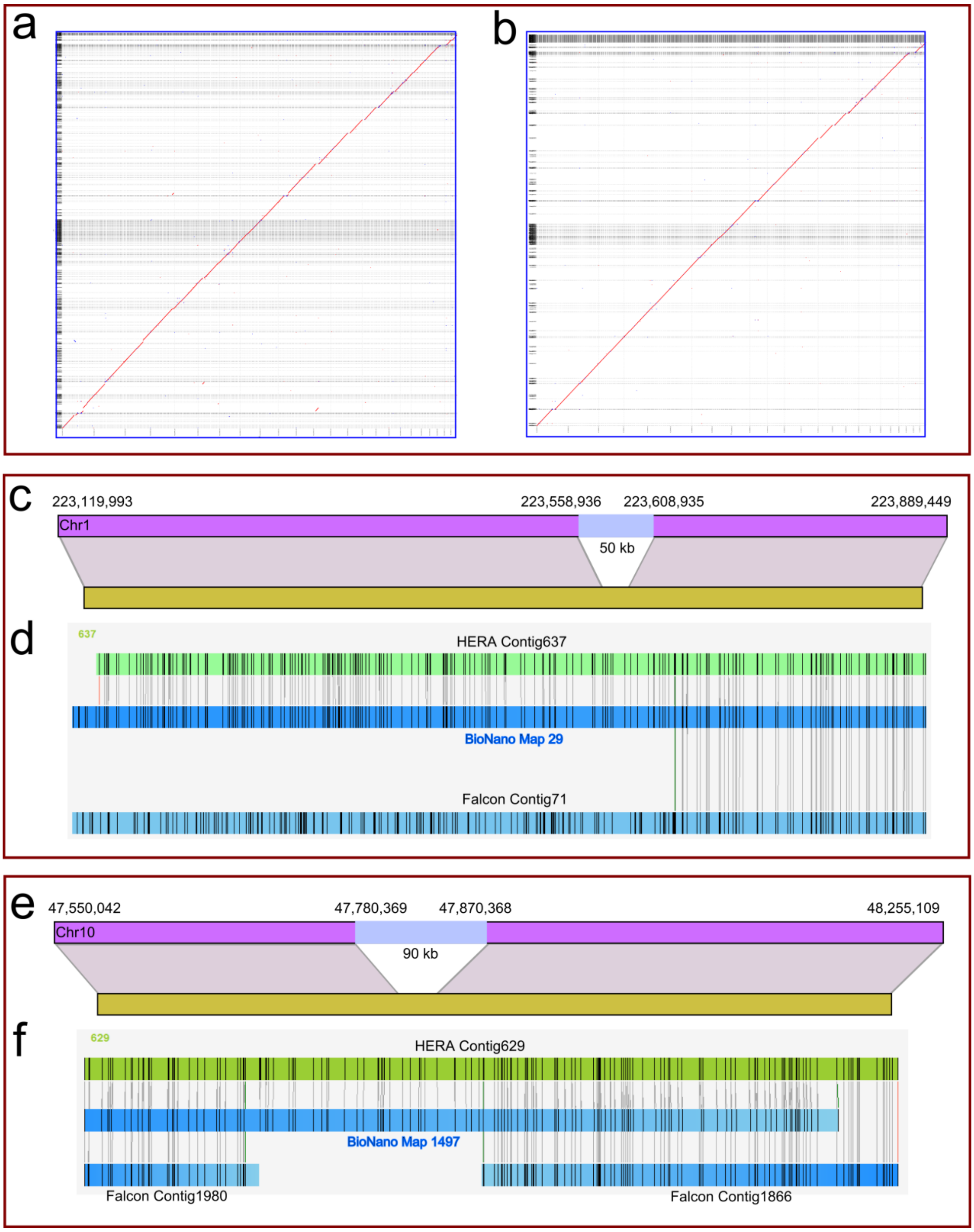

Supplementary Figure 8: Examples of potential gap closing in GRCh38 reference genome by HERA.

(**a**) The alignment of HX1_FALCON to GRCH38. (**b**) The alignment of HX1_HERA1 to GRCH38. (**c**)(**d**) A gap on GRCh38 chromosome 1 could be closed based on sequence of HX1_HERA1. HERA correctly assembled a whole region that has a missing sequence in HX1_FALCON. The gap in GRCH38 is within the missing sequence of HX1_FALCON. (**e**)(**f**) Another gap on GRCh38 chromosome 10 could be closed using the sequence in HX1_HERA1. HERA correctly assembled the region that contained two contigs and a missing sequence in HX1_Falcon. The gap on GRCh38 chromosome 10 is within the missing sequence of HX1_FALCON.

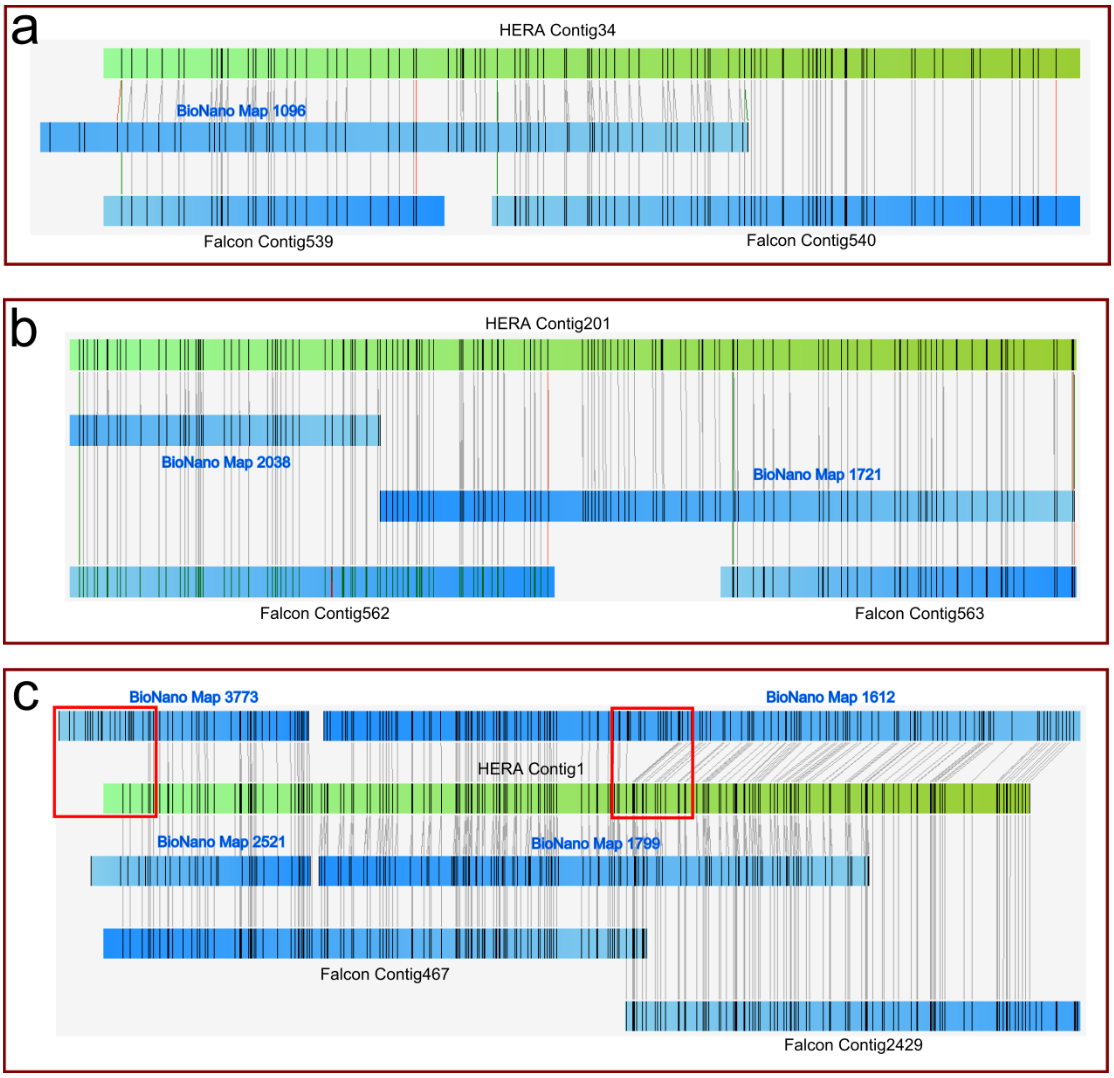

Supplementary Figure 9: The examples of potential falsely anchored sequences in either chromosome X or chromosome Y in GRCh38.

The HERA contigs Contig34, Contig201 and Contig1 were each partially aligned to GRCh38 chromosomes X and Y with some segments matching to only one chromosome and some other segments matching to both chromosomes (Supplementary Table 5). (**a**)(**b**) A single group of BioNano map completely covered the HERA contigs in each case which proved their correctness. (**c**) A single HERA contig Contig1 matched to two groups of BioNano maps. One map is consistent with Contig1 while the other one contained indels and overhangs compared with Contig1, suggesting that the corresponding sequences in GRCh38 chromosomes X and Y were misassembled or mispositioned.

Supplementary Tables

Supplementary Table 1: Summary of raw and corrected PacBio data

Genome	Type	Read num	N50 (bp)	Average (bp)	Total (Gb)	Depth of Coverage (x)
**R498**	Raw	6085344	11201	7766	47	118
	Corrected	2013831	8424	8666	17	43
**B73**	Raw	13698313	16699	12206	81	76
	Corrected	5304672	32775	15358	167	37
**HX1**	Raw	44207919	12134	6990	309	103
	Corrected	9972749	12911	11912	118	39
**Pinku1**	Raw	5249615	10083	6941	36	80
	Corrected	2353782	9369	6946	16	35

The R498 SMRT reads were self-corrected by PBcR with default parameters. The SMRT reads of B73, HX1 amd Pinku1 were self-corrected by CANU with default parameters.

Supplementary Table 2: Summary of hybrid scaffolds with BioNano maps.

Genome	Method	Num	N50 (Mb)	Max len (Mb)	Total len (Mb)	Gap num	Gap len (Mb)
**R498**	BioNano+CANU	105	5.67	18.25	388.9	334	5.91
	BioNano+HERA	32	17.51	32.2	390.2	28	1.16
**B73**	BioNano+PBcR	319	10.2	45.88	2060.3	2002	43.07
	BioNano+HERA	68	107.5	194.6	2110	18	0.98
**HX1**	BioNano+FALCON	325	24.05	83.66	2724.4	476	39.37
**Pinku1**	BioNano+PBcR	550	5.43	15.04	451.9	781	13.29
	BioNano+HERA	22	51.77	61.99	453.7	8	0.49

Note that each genome included unfiltered contaminated sequences.

Supplementary Table 3: Summary of sequence alignment of rice BACs to HERA assembled regions and CANU assembled regions.

GenBank ID	BAC Clone	Length (bp)	HERA Sequence Identity (%)	HERA Sequence Len (bp)	CANU Sequence Identity (%)	CANU Sequence Len (bp)	BioNano Map Support
**AL731599.2**	OSJNBa0053B21	151,936	99.43	51,330	99.30	100,606	Yes
**AL606690.3**	OSJNBa0060D06	151,506	99.41	71,907	99.37	79,599	Yes
**AL731594.5**	OSJNBa0032N05	159,486	99.28	89,879	99.47	69,607	Yes
**AL662972.3**	OSJNBa0063G07	157,622	99.65	56,640	99.67	100,640	Yes
**AP003372.2**	OJ1014_G12	158,830	99.63	53,308	99.61	105,522	Yes
**AL731601.3**	OSJNBa0044M19	188,317	99.27	49,356	99.19	138,961	Yes
**AL663022.4**	OSJNBa0054D14	160,587	99.83	65,769	99.76	94,818	Yes
**AL662958.3**	OSJNBa0019D11	163,039	99.37	43,740	99.38	119,299	Yes

Supplementary Table 4: The comparison of HERA constructed B73 genome and B73 RefGen_v4 reference genome

Chr	Chr_len* (B73_HERA1)	Chr_len* (RefGen_v4)	Gap_num (B73_HERA1)	Gap_num (RefGen_v4)	Gap_len** (B73_HERA1)	Gap_len** (RefGen_v4)	Gene_num (B73_HERA1)	Gene_num (RefGen_v4)
**Chr1**	308,056,010	303,355,124	15	348	158,216	3,686,593	7,009	6,948
**Chr2**	242,550,780	240,531,092	7	292	6,135	3,911,184	5,817	5,780
**Chr3**	236,649,599	233,293,116	6	276	51,686	2,374,718	5,000	4,945
**Chr4**	247,697,134	242,944,880	11	276	457,490	4,049,725	5,167	5,118
**Chr5**	223,598,723	220,830,651	10	281	197,463	3,071,589	5,467	5,293
**Chr6**	173,718,017	169,277,604	10	195	93,249	4,755,566	4,138	4,049
**Chr7**	178,308,893	180,151,203	5	238	1,299	2,230,339	3,781	3,762
**Chr8**	180,052,061	178,721,167	6	230	1,200	2,401,470	4,499	4,263
**Chr9**	160,890,507	157,329,761	2	213	400	2,440,021	3,650	3,590
**Chr10**	152,387,480	149,170,640	4	174	22,811	1,811,674	3,288	3,253
**Total**	2,103,909,204	2,075,605,238	76	2,523	989,949	30,732,879	47,816	47,001

*The total length of the contigs, not including the length of the gaps. **The gap length indicates the Ns on each chromosome, including known and unknown length of gaps, so the real length is unknown.

Supplementary Table 5: Summary of the potential indels in the HERA assembled B73 genome identified by comparison with BioNano genome maps

Chr ID	Start	End	Genome Map ID	Start	End	Len Diff (bp)	T	S
**1**	4,551,618	4,583,619	220	1,773,106	1,782,926	22,181	D	P
**1**	21,627,254	21,631,714	166	3,167,754	3,151,181	12,112	I	P
**1**	24,612,728	24,646,932	166	177,045	169,720	26,880	D	P
**1**	25,812,888	25,836,266	750	255,228	250,242	18,393	D	P
**1**	51,137,480	51,139,100	2,511	844,509	878,954	32,825	I	P
**1**	56,807,880	56,831,828	1,987	1,475,687	1,480,927	18,708	D	P
**1**	62,335,496	62,360,336	1,034	5,294,871	5,293,142	23,111	D	P
**1**	93,187,936	93,210,640	429	1,412,803	1,408,391	18,292	D	P
**1**	104,527,680	104,542,784	251	2,734,151	2,729,686	10,640	D	P
**1**	119,389,288	119,405,216	164	337,893	343,751	10,070	D	P
**1**	138,412,576	138,450,912	3	11,377,289	11,203,336	135,616	I	P
**1**	139,730,288	139,731,920	3	9,928,532	9,914,203	12,696	I	P
**1**	156,598,608	156,627,744	1,723	126,192	142,466	12,863	D	P
**1**	193,006,832	193,007,432	565	1,019,188	986,209	32,378	I	P
**1**	210,659,328	210,662,320	823	803,724	817,539	10,823	I	P
**1**	220,322,912	220,327,280	229	3,996,593	3,982,071	10,153	I	P
**1**	240,802,928	240,832,896	331	1,730,312	1,717,058	16,714	D	P
**1**	252,363,456	252,363,920	107	2,705,339	2,598,686	106,188	I	P

**1**	252,594,048	252,743,504	107	2,368,525	2,345,568	126,499	D	P
**1**	41,562,720	41,563,328	411	1,603,771	1,614,576	10,196	I	H
**1**	138,157,472	138,223,248	382	2,178,795	2,317,594	73,023	I	H
**1**	171,121,920	171,144,800	4	5,743,210	5,709,924	10,405	I	H
**1**	228,309,440	228,312,176	526	703,060	785,239	79,443	I	H
**1**	289,428,176	289,430,656	1,887	1,011,956	956,126	53,350	I	H
**1**	300,947,760	300,964,384	182	1,685,001	1,727,084	25,459	I	H
**1**	301,014,224	301,056,368	182	1,776,674	1,793,305	25,514	D	H
**2**	80,015,080	80,037,328	2,018	766,315	763,668	19,601	D	P
**2**	115,079,080	115,081,864	539	547,781	589,300	38,735	I	P
**2**	118,257,728	118,283,536	3,766	77,400	65,562	13,970	D	P
**2**	121,507,128	121,527,920	540	1,119,071	1,110,016	11,737	D	P
**2**	162,410,144	162,442,496	385	955,771	946,138	22,720	D	P
**2**	165,856,768	165,901,504	3,689	1,021,076	1,046,247	19,565	D	P
**2**	171,579,360	171,606,688	632	741,265	734,099	20,163	D	P
**2**	174,924,608	174,965,264	2,081	838,106	834,500	37,050	D	P
**2**	178,477,664	178,478,368	1,238	2,278,098	2,257,099	20,295	I	P
**2**	203,955,328	203,990,784	547	165,508	159,071	29,019	D	P
**2**	215,171,360	215,193,184	1,833	1,484,862	1,482,038	19,000	D	P
**2**	215,240,032	215,242,896	1,833	1,434,920	1,421,056	11,000	I	P
**2**	75,289,872	75,312,208	507	62,335	95,328	10,656	I	H
**2**	96,765,560	96,790,704	105	802,142	414,768	362,230	I	H
**2**	151,660,256	151,665,200	1,852	1,069,616	1,085,124	10,563	I	H
**2**	217,557,552	217,565,856	566	1,145,287	1,126,764	10,219	I	H
**2**	232,741,080	232,760,960	459	1,927,116	1,921,680	14,445	D	H
**3**	3,380,351	3,386,144	172	363,486	391,809	22,530	I	P
**3**	3,977,446	4,008,238	515	1,542,193	1,535,613	24,213	D	P
**3**	27,530,224	27,530,692	110	773,158	759,412	13,277	I	P
**3**	75,733,656	75,740,384	1,884	1,460,066	1,488,359	21,565	I	P
**3**	87,992,800	88,008,048	5	6,960,711	6,865,271	80,192	I	P
**3**	105,506,856	105,513,984	145	1,475,777	1,458,055	10,593	I	P
**3**	128,586,280	128,611,656	1,601	2,083,991	2,076,791	18,177	D	P
**3**	144,510,320	144,530,368	241	2,644,468	2,652,100	12,416	D	P
**3**	150,990,464	151,009,600	198	836,144	831,175	14,168	D	P
**3**	163,183,568	163,185,296	9	6,820,180	6,796,128	22,323	I	P
**3**	164,899,120	164,904,704	9	5,083,559	5,067,322	10,653	I	P
**3**	166,566,416	166,571,040	9	3,406,828	3,391,543	10,660	I	P
**3**	166,605,776	166,625,520	9	3,357,138	3,347,476	10,083	D	P
**3**	166,712,560	166,719,152	9	3,260,023	3,229,787	23,644	I	P
**3**	173,180,576	173,203,280	260	3,140,324	3,145,029	18,000	D	P
**3**	195,414,128	195,445,216	70	2,761,641	2,756,026	25,473	D	P
**3**	226,633,904	226,647,936	80	1,350,763	1,317,457	19,274	I	P
**3**	44,842,672	44,853,056	24	3,306,079	3,272,171	23,524	I	H
**3**	47,012,008	47,186,684	24	1,078,940	1,073,603	169,340	D	H

**3**	98,998,704	98,999,304	190	1,341,124	1,351,769	10,045	I	H
**3**	193,276,016	193,276,528	2,136	742,063	722,278	19,273	I	H
**4**	1,854,630	1,857,412	69	2,683,689	2,667,071	13,835	I	P
**4**	1,880,434	1,906,492	69	2,643,986	2,631,738	13,809	D	P
**4**	2,167,096	2,172,263	69	2,383,383	2,289,294	88,922	I	P
**4**	2,176,326	2,180,783	69	2,284,903	2,162,138	118,308	I	P
**4**	3,678,850	3,679,810	69	672,211	497,768	173,483	I	P
**4**	4,852,205	4,854,760	1,249	654,041	717,791	61,194	I	P
**4**	63,852,460	63,854,028	265	322,095	405,966	82,303	I	P
**4**	72,974,656	72,999,632	124	555,341	550,141	19,777	D	P
**4**	78,581,704	78,602,320	257	206,748	212,517	14,848	D	P
**4**	131,888,392	131,895,392	319	1,211,161	1,239,535	21,374	I	P
**4**	163,438,512	163,477,056	3,785	2,658,465	2,641,106	21,185	D	P
**4**	169,117,712	169,120,992	55	1,631,353	1,756,230	121,597	I	P
**4**	182,853,536	182,884,016	27	4,782,374	4,779,095	27,202	D	P
**4**	183,884,336	183,885,120	27	3,783,655	3,761,355	21,516	I	P
**4**	184,491,936	184,517,344	27	3,153,613	3,140,001	11,797	D	P
**4**	77,660,416	77,661,056	597	788,279	712,517	75,121	I	H
**4**	89,165,984	89,190,736	622	4,370,581	4,335,326	10,502	I	H
**4**	107,775,568	107,777,352	591	2,578,609	2,534,498	42,326	I	H
**4**	108,233,520	108,330,112	591	2,084,649	1,961,646	26,411	I	H
**4**	192,258,336	192,261,408	355	798,243	824,332	23,016	I	H
**5**	25,237,346	25,253,550	131	3,057,566	3,054,006	12,645	D	P
**5**	43,779,696	43,808,892	202	896,092	898,790	26,498	D	P
**5**	68,062,976	68,067,472	289	428,482	550,603	117,624	I	P
**5**	71,643,856	71,654,600	2,468	348,209	314,223	23,241	I	P
**5**	79,141,352	79,210,400	12	3,109,193	3,143,777	34,464	D	P
**5**	105,676,600	105,697,496	812	37,258	88,631	30,477	I	P
**5**	115,897,520	115,921,344	204	2,314,844	2,311,563	20,543	D	P
**5**	116,575,032	116,594,712	204	1,659,122	1,628,644	10,797	I	P
**5**	124,591,816	124,593,544	222	2,588,328	2,689,645	99,588	I	P
**5**	124,596,936	124,597,568	222	2,693,160	2,792,134	98,341	I	P
**5**	124,619,472	124,621,264	222	2,813,962	2,924,990	109,236	I	P
**5**	154,451,984	154,482,144	2,593	383,267	373,410	20,303	D	P
**5**	168,284,720	168,309,280	1,841	148,005	153,778	18,788	D	P
**5**	212,519,472	212,540,832	21	3,728,278	3,725,938	19,021	D	P
**5**	212,559,024	212,582,384	21	3,708,147	3,616,755	68,031	I	P
**5**	212,680,800	212,740,352	21	3,517,947	3,439,702	18,693	I	P
**5**	215,348,960	215,516,336	21	833,382	822,781	156,775	D	P
**5**	215,516,336	215,578,256	21	822,781	820,340	59,479	D	P
**5**	11,658,413	11,673,350	297	1,212,306	1,251,576	24,333	I	H
**5**	195,065,600	195,066,112	536	663,428	675,297	11,356	I	H
**5**	203,274,832	203,283,904	1,108	227,000	207,083	10,845	I	H
**6**	11,655,017	11,664,047	157	3,378,070	3,474,455	87,355	I	P

**6**	11,808,197	11,960,196	157	3,845,183	3,953,565	43,618	D	P
**6**	12,419,332	12,582,017	157	4,275,583	4,486,129	47,861	I	P
**6**	12,594,371	12,710,119	157	4,498,076	4,710,764	96,939	I	P
**6**	12,807,213	12,852,586	157	4,809,283	4,906,676	52,020	I	P
**6**	82,638,008	82,658,352	781	1,034,455	1,002,957	11,154	I	P
**6**	91,890,080	91,919,904	2,517	486,089	492,269	23,645	D	P
**6**	102,223,040	102,334,112	549	1,072,124	1,012,854	51,802	D	P
**6**	102,410,080	102,422,424	549	937,203	820,892	103,967	I	P
**6**	102,498,584	102,504,280	549	744,528	659,940	78,892	I	P
**6**	119,670,536	119,675,088	16	5,137,337	5,197,908	56,019	I	P
**6**	131,105,208	131,120,336	191	2,250,796	2,253,303	12,621	D	P
**6**	158,316,032	158,327,056	1,120	143,336	120,716	11,595	I	P
**6**	25,069,964	25,113,676	1,321	136,039	351,780	172,029	I	H
**6**	78,605,848	78,609,704	5,841	159,753	232,959	69,349	I	H
**6**	90,939,000	90,942,816	1,113	66,909	165,515	94,789	I	H
**6**	138,975,360	138,976,960	23	1,765,129	1,782,658	15,929	I	H
**6**	144,021,424	144,026,192	519	1,183,658	1,168,815	10,074	I	H
**6**	145,947,072	145,961,856	3,609	749,908	775,505	10,812	I	H
**7**	50,087,012	50,113,532	465	524,626	526,794	24,353	D	P
**7**	91,966,704	91,973,616	30	1,620,466	1,747,711	120,333	I	P
**7**	91,975,896	91,976,488	30	1,749,893	1,907,576	157,090	I	P
**7**	117,633,816	117,659,008	1,096	1,182,418	1,187,392	20,219	D	P
**7**	118,997,800	118,998,344	1,096	2,524,095	2,658,204	133,564	I	P
**7**	118,998,344	119,008,712	1,096	2,658,204	2,761,083	92,511	I	P
**7**	134,448,192	134,481,360	112	261,698	266,819	28,048	D	P
**7**	40,952,836	40,957,768	914	278,159	293,534	10,442	I	H
**7**	53,857,840	53,863,560	605	665,559	638,953	20,885	I	H
**7**	58,093,084	58,094,880	31	1,870,637	2,105,208	232,775	I	H
**7**	153,846,240	153,906,896	593	1,495,884	1,449,869	14,642	D	H
**7**	170,307,392	170,310,640	78	1,244,441	1,294,574	46,884	I	H
**8**	8,013,767	8,029,805	113	3,831,026	3,833,259	13,806	D	P
**8**	13,753,276	13,775,711	1,436	1,777,472	1,786,409	13,499	D	P
**8**	31,443,820	31,485,556	375	512,048	526,765	27,019	D	P
**8**	39,075,256	39,131,980	14	4,545,850	4,561,073	41,502	D	P
**8**	78,990,224	79,031,080	40	2,078,496	2,109,093	10,259	D	P
**8**	119,585,184	119,596,248	139	2,133,217	2,110,471	11,681	I	P
**8**	121,115,224	121,138,704	139	594,208	592,396	21,669	D	P
**8**	144,940,496	144,954,448	1,070	202,738	205,706	10,985	D	P
**8**	151,482,624	151,507,976	3,595	1,690,831	1,682,697	17,219	D	P
**8**	161,398,224	161,415,648	1,989	1,706,424	1,751,898	28,049	I	P
**8**	9,006,658	9,010,353	281	940,623	957,455	13,137	I	H
**8**	48,852,668	48,887,288	2,701	386,666	76,662	275,384	I	H
**8**	80,450,664	80,454,464	456	1,105,823	1,091,173	10,850	I	H
**8**	81,370,656	81,371,264	456	181,671	106,727	74,335	I	H

**8**	99,272,728	99,273,424	2,348	296,671	307,479	10,111	I	H
**8**	123,695,512	123,697,472	1,662	2,784,927	2,740,717	42,249	I	H
**9**	11,251,617	11,285,410	684	266,574	262,445	29,664	D	P
**9**	16,744,848	16,746,469	755	757,792	730,936	25,234	I	P
**9**	28,676,688	28,681,580	160	1,457,341	1,509,112	46,879	I	P
**9**	36,252,288	36,283,340	1,567	4,217,106	4,212,000	25,947	D	P
**9**	61,730,944	61,734,020	200	2,808,688	2,732,349	73,263	I	P
**9**	62,066,764	62,067,252	200	2,400,298	2,363,443	36,367	I	P
**9**	65,387,348	65,432,912	2,623	490,378	510,689	25,253	D	P
**9**	74,886,064	74,889,224	336	1,239,754	1,164,654	71,939	I	P
**9**	75,011,464	75,027,944	336	1,042,536	946,069	79,987	I	P
**9**	75,033,032	75,087,832	336	940,778	859,604	26,374	I	P
**9**	75,448,512	75,508,456	336	506,186	431,465	14,776	I	P
**9**	129,592,212	129,594,832	132	1,740,084	1,697,680	39,783	I	P
**9**	130,020,520	130,038,248	132	1,272,396	1,190,430	64,238	I	P
**9**	135,718,400	135,734,016	63	3,413,853	3,387,642	10,595	I	P
**9**	136,130,080	136,140,864	63	2,991,265	2,969,798	10,683	I	P
**9**	151,009,920	151,017,088	8	5,497,197	5,536,397	32,032	I	P
**9**	46,279,352	46,282,512	441	1,260,914	1,156,385	101,369	I	H
**9**	58,256,360	58,256,968	858	400,081	244,912	154,561	I	H
**10**	608,982	634,423	444	831,218	822,535	16,759	D	P
**10**	4,573,274	4,574,790	57	3,101,099	3,124,600	21,985	I	P
**10**	5,247,608	5,268,361	57	3,798,266	3,807,023	11,996	D	P
**10**	6,961,628	6,962,966	133	2,555,972	2,511,536	43,097	I	P
**10**	9,093,998	9,121,761	133	382,448	380,310	25,625	D	P
**10**	38,141,936	38,177,644	294	1,223,445	1,209,517	21,781	D	P
**10**	55,834,056	55,859,336	208	2,228,581	2,233,614	20,247	D	P
**10**	93,578,528	93,603,784	879	424,821	433,472	16,605	D	P
**10**	106,456,608	106,460,392	851	486,445	533,904	43,675	I	P
**10**	116,794,288	116,817,752	323	1,461,361	1,473,163	11,662	D	P
**10**	119,931,664	119,937,816	2,503	711,432	790,697	73,113	I	P
**10**	136,809,600	136,812,784	625	1,159,427	1,196,129	33,517	I	P
**10**	137,615,216	137,651,472	1,456	426,281	437,216	25,322	D	P
**10**	27,041,284	27,098,164	76	1,468,306	1,599,235	74,048	I	H
**10**	49,514,504	49,543,636	154	2,800,658	2,741,790	29,736	I	H
**10**	58,877,600	58,878,100	2,291	1,459,724	1,448,138	11,085	I	H
**10**	94,431,568	94,448,688	1,940	315,801	343,547	10,625	I	H

Abbreviations: T, variation type; I, insertion; D, deletion; S, software; P, PBcR; H, HERA.

Supplementary Table 6: The alignment of HERA constructed HX1 contigs to GRCh38 chromosomes X and Y

Query	Query Start	Query End	Chr	Ref ID	Ref Start (bp)	Ref End (bp)	Identity (%)
Contig1	0	83,694	X	NC_000023.11	64,927,911	65,011,805	99.48
	83,835	651,343	Y	NC_000024.10	52,624,446	53,194,126	98.54
	652,064	922,298	X	NC_000023.11	65,583,723	65,855,205	98.76
	833,077	1,461,238	Y	NC_000024.10	53,383,205	54,016,181	98.19
	1,382,224	1,675,903	X	NC_000023.11	66,406,295	66,701,315	98.72
	1,675,899	1,798,927	X	NC_000023.11	66,685,587	66,809,174	98.91
Contig34	40,275	192,785	X	NC_000023.11	1,764,535	1,919,981	97.17
	40,275	192,785	Y	NC_000024.10	1,764,535	1,919,981	97.17
	220,908	364,992	Y	NC_000024.10	2,133,035	2,277,067	98.53
	220,908	364,791	X	NC_000023.11	2,133,035	2,276,863	98.53
	366,326	463,514	Y	NC_000024.10	2,282,303	2,374,747	93.42
	463,226	1,025,152	X	NC_000023.11	2,376,715	2,939,095	99.15
Contig201	0	503,301	X	NC_000023.11	154,800,564	155,304,571	99.63
	535,355	703,527	X	NC_000023.11	155,336,693	155,504,551	98.92
	653,061	999,112	X	NC_000023.11	155,453,983	155,803,824	98.53
	1,001,253	1,219,112	X	NC_000023.11	155,803,260	156,021,579	99.45
	1,001,253	1,219,112	Y	NC_000024.10	56,989,780	57,208,099	99.45

Supplementary Table 7: Summary of comparing the HERA assembled HX1 regions to other human genomes.

Genome Name	GenBank Accession	Contig N50 (bp)	Genome size (bp)	Flanking Sequence Identity (%)	HERA Sequence Covered (%)	HERA Sequence Identity (%)	HERA Sequence Covered Num
**GRCh38.p13**	PRJNA31257	57,879,411	3,099,706,404	99.08	85.89	99.12	826
**CHM1**	PRJNA246220	34,007,701	2,989,427,264	99.18	83.55	99.14	814
**HG00514**	PRJNA300843	29,396,877	2,864,989,099	99.35	79.64	99.28	749
**CHM13**	PRJNA369439	29,260,714	2,875,999,248	98.96	79.61	99.15	732
**NA19240**	PRJNA288807	29,140,631	2,866,746,472	99.22	79.88	99.20	774
**HG00733**	PRJNA483067	26,292,878	2,893,114,812	99.25	81.19	99.16	758
**HG02059**	PRJNA339726	25,293,151	2,898,275,003	99.37	80.57	99.30	759
**HG02818**	PRJNA339722	22,499,404	2,875,770,361	99.27	76.78	99.19	707
**AK1_v2**	PRJNA298944	18,080,262	2,866,867,749	99.36	77.94	99.30	720
**HG002**	PRJNA529552	16,145,298	2,957,656,065	98.40	70.70	98.97	557

The flanking sequences in HX1 were assembled by FALCON. There are a total of 1,192 HERA assembled regions in HX1, with a total length of 48.47 Mb, and a max length of 321,559 bp. Both types of sequences were aligned to each genome, and the average sequence identity and coverage were calculated. Note that the three Asian genomes AK1, HG02059 and HG00514 have the highest sequence identity with HX1 among the 10 genomes, consistent with their closer relationship to HX1 than other genomes.
